# Development and acceptability testing of a decision aid for considering whether to reduce antipsychotics in individuals with stable schizophrenia

**DOI:** 10.1002/npr2.12366

**Published:** 2023-07-14

**Authors:** Yumi Aoki, Yoshikazu Takaesu, Kentaro Matsui, Takahiro Tokumasu, Hideaki Tani, Yoshiteru Takekita, Tetsufumi Kanazawa, Taishiro Kishimoto, Seiichiro Tarutani, Naoki Hashimoto, Hiroyoshi Takeuchi, Kazuo Mishima, Ken Inada

**Affiliations:** ^1^ Department Psychiatric and Mental Health Nursing, Graduate School of Nursing St. Luke's International University Tokyo Japan; ^2^ Department of Neuropsychiatry Kyorin University School of Medicine Tokyo Japan; ^3^ Department of Neuropsychiatry, Graduate School of Medicine University of the Ryukyus Okinawa Japan; ^4^ Department of Clinical Laboratory National Center Hospital, National Center of Neurology and Psychiatry Tokyo Japan; ^5^ Department of Psychiatry Showa University Northern Yokohama Hospital Kanagawa Japan; ^6^ Department of Neuropsychiatry Keio University School of Medicine Tokyo Japan; ^7^ Department of Neuropsychiatry, Faculty of Medicine Kansai Medical University Osaka Japan; ^8^ Department of Neuropsychiatry, Faculty of Medicine Osaka Medical and Pharmaceutical University Osaka Japan; ^9^ Hills Joint Research Laboratory for Future Preventive Medicine and Wellness Keio University School of Medicine Tokyo Japan; ^10^ Department of Psychiatry Shin‐Abuyama Hospital, Osaka Institute of Clinical Psychiatry Osaka Japan; ^11^ Department of Psychiatry Hokkaido University Graduate School of Medicine Hokkaido Japan; ^12^ Department of Neuropsychiatry Akita University Graduate School of Medicine Akita Japan; ^13^ Department of Psychiatry, School of Medicine Kitasato University Kanagawa Japan

**Keywords:** acceptability, antipsychotics, decision aid, schizophrenia, shared decision‐making

## Abstract

**Aim:**

Continued antipsychotic treatment is the key to preventing relapse. Maintenance antipsychotic monotherapy and optimal dose use are recommended for individuals with stable schizophrenia because of their undesirable effects. Decision aids (DAs) are clinical conversation tools that facilitate shared decision‐making (SDM) between patients and health‐care providers. This study aimed to describe the development process and results of acceptability testing of a DA for individuals with stable schizophrenia, considering (i) whether to continue high‐dose antipsychotics or reduce to the standard dose and (ii) whether to continue two antipsychotics or shift to monotherapy.

**Methods:**

A DA was developed according to the guidelines for the appropriate use of psychotropic medications and International Patient Decision Aid Standards (IPDAS). First, a DA prototype was developed based on a previous systematic review and meta‐analysis conducted for identifying the effects of continuing or reducing antipsychotic treatment. Second, mixed‐method survey was performed among individuals with schizophrenia and health‐care providers to modify and finalize the DA.

**Results:**

The DA consisted of an explanation of schizophrenia, options to continue high‐dose antipsychotics or reduce to the standard dose, options to continue two antipsychotics or shift to monotherapy, pros and cons of each option, and a value‐clarification worksheet for each option. The patients (*n* = 20) reported acceptable language use (75%), adequate information (75%), and well‐balanced presentation (79%). Health‐care providers (*n* = 20) also provided favorable overall feedback. The final DA covered six IPDAS qualifying criteria.

**Conclusion:**

A DA was successfully developed for schizophrenia, considering whether to reduce antipsychotics, which can be used in the SDM process.

## Abstract 2

A DA was successfully developed for schizophrenia, considering whether to reduce antipsychotics, which can be used in the SDM process.

## Graphical abstract

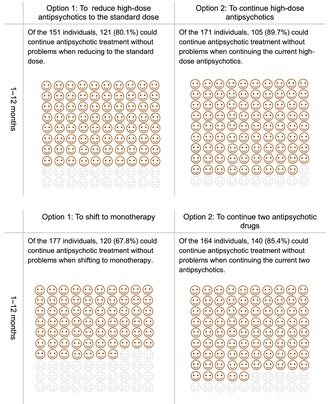

## 1 INTRODUCTION

Schizophrenia is a psychiatric disorder characterized by repeated relapse of psychotic episodes.^1^ Approximately 0.3%–0.7% of individuals worldwide are diagnosed with schizophrenia.^2^ Individuals with schizophrenia suffer from significant distress and impairment in personal, family, social, educational, occupational, and other important areas of life.^3^ Therefore, continued improvements in the treatment for schizophrenia are crucial, and maintenance treatment using antipsychotics is particularly important to prevent relapse.^4^, ^5^ Although continued antipsychotic treatment is essential for preventing relapse, antipsychotics have undesirable effects, including extrapyramidal symptoms,^6^ neurocognitive impairments,^7^, ^8^, ^9^ and sudden cardiac deaths,^10^ which are at least partially dose dependent.^11^ Accordingly, it is clinically important to minimize long‐term antipsychotic use. Several trials have been conducted to reduce the use of antipsychotics. For example, a meta‐analysis of six randomized controlled trials (RCTs) examining a switch from antipsychotic polypharmacy to monotherapy and remaining on antipsychotic polypharmacy showed a significant difference in study discontinuation due to all causes, whereas no significant differences in relapse, psychopathology, neurocognition, extrapyramidal symptoms, or body weight/body mass index (BMI) between the two groups were noted.^12^ Another meta‐analysis of 18 RCTs investigating antipsychotic dose reduction in schizophrenia revealed that the relapse rate was significantly higher in the reduction group than in the maintenance group, whereas neurocognition was significantly improved.^13^

The result from these meta‐analyses raises another clinical question: how should we apply the evidence from these meta‐analyses to clinical practice? Understanding how individuals receiving treatment perceive these results is important. This indicates that sharing treatment decisions with patients is essential. This is because even if there is a proven intervention, without shared decision‐making (SDM), evidence‐based medicine can turn into evidence tyranny.^14^

SDM has gained attention in psychiatric disorders and other physical disorders.^15^ Decision aids (DAs) are decision‐support tools that help stakeholders, including patients and health‐care providers, participate in the SDM process. Thus, DAs help clarify health‐care decisions that need to be considered, show relevant information and outcomes of related options, and explore patients' own preferences and values.^16^ Some DAs for schizophrenia have been developed, such as a booklet used in a psychiatric acute ward^17^ and a digital tool for first episode of psychosis,^18^ which aimed to facilitate SDM in the initial phase of treatment. However, to the best of our knowledge, no DA is currently available for individuals with stable schizophrenia who are considering reducing antipsychotics in maintenance treatment.

This study aimed to describe the development process and results of the acceptability testing of a DA for individuals with stable schizophrenia, considering (i) whether to continue high‐dose antipsychotics or reduce to the standard dose and (ii) whether to continue two antipsychotics or shift to monotherapy.

## 2 METHODS

### 2.1 Study design

**FIGURE 1:**
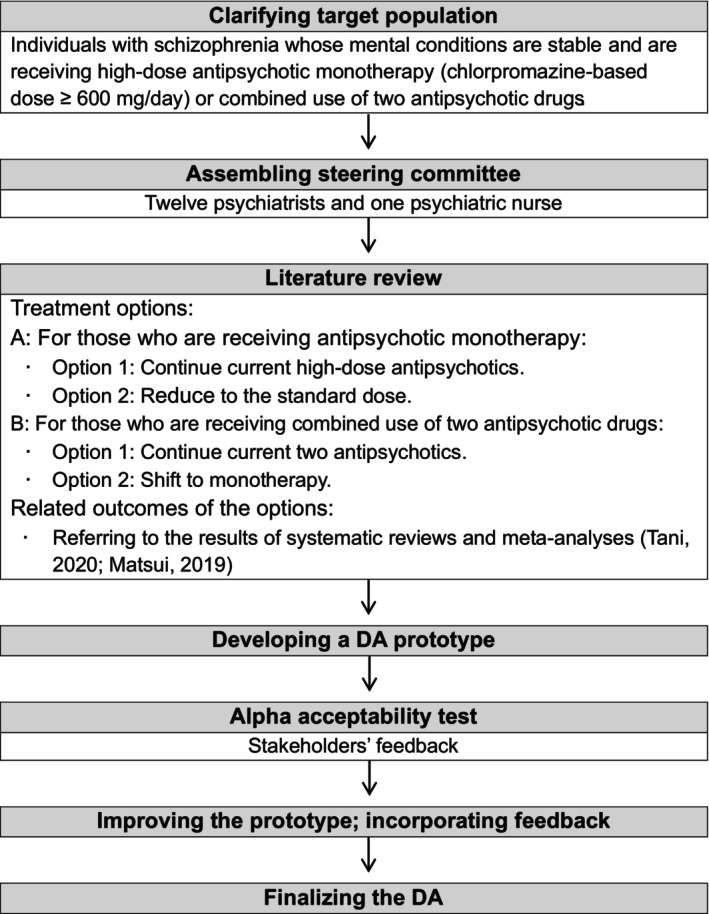
Development process of a DA for schizophrenia considering whether to reduce antipsychotics based on the framework of Coulter et al.^22^

We developed a DA according to the guidelines for appropriate use of psychotropic medications,^19^ Ottawa Decision Support Framework,^20^ and International Patient Decision Aid Standards (IPDAS)^21^ (Figure 1). The IPDAS are an evidence‐based criteria that provide the development process and contents of DAs.^21^ The process of DA development includes (1) clarifying the target population and their decisional needs, (2) gathering a steering committee of specialists, (3) reviewing previous studies to determine the treatment options and its outcomes, (4) developing a prototype of the DA, (5) acceptability testing (alpha test) of the prototype among stakeholders who are not involved in the DA development, (6) improving the prototype following the results of the acceptability testing to finalize the DA, and (7) field testing (beta test) of the DA to determine its effectiveness in actual clinical settings.^22^

### 2.2 Target population

In this study, the DA targeted individuals with schizophrenia whose conditions are stable with undergoing antipsychotic treatment: (i) high‐dose antipsychotic monotherapy (chlorpromazine‐based dose ≥600 mg/day) or (ii) combined use of two antipsychotic drugs. The DA did not target those who were receiving antipsychotic treatment but still suffered from active symptoms of schizophrenia. Furthermore, the DA excluded those receiving more than two antipsychotics as combined therapy.

### 2.3 Steering committee

A steering committee consisting of experts on schizophrenia treatment (all listed as authors) and DA methodology was gathered. The committee included 12 psychiatrists who regularly attend to individuals with schizophrenia and a psychiatric nurse with sufficient knowledge about SDM for those with mental health conditions^15^, ^23^ and experience developing DAs in psychiatry.^24^, ^25^, ^26^

### 2.4 Literature review

#### 2.4.1 Determining pros and cons of the options

Previous studies were reviewed to identify schizophrenia as the target health condition. We then identified the pros and cons of two options regarding each decision: (i) to continue high‐dose antipsychotics or reduce to the standard dose (for antipsychotic monotherapy) and (ii) to continue polypharmacy or shift to monotherapy (for the combined use of two antipsychotic drugs). Moreover, we searched for relevant information such as chlorpromazine‐equivalent doses for antipsychotics, side effects of antipsychotics, concept of recovery, and coping strategies to promote recovery.

#### 2.4.2 Determining related outcomes of the options

First, regarding the consequences of the two options for monotherapy—to continue high‐dose antipsychotics or reduce to the standard dose—we cited the results of an additional subgroup analysis of previously conducted meta‐analysis.^13^ The subgroup analysis focused on trials that compared study discontinuation due to all causes between those who continued high‐dose antipsychotics and those who reduced it to the standard dose.^13^ Second, regarding the consequences of two options for the combined use of two antipsychotics—to continue polypharmacy or shift to monotherapy—we referred the results of a previous systematic review and meta‐analysis.^12^ The meta‐analysis focused on trials that compared study discontinuation due to all causes on those who continued polypharmacy (two antipsychotic drugs) and those who shifted to monotherapy.^12^

### 2.5 Prototype development

Based on the results of our literature review, we developed a DA prototype according to the IPDAS criteria.^21^ The prototype was written in Japanese.

### 2.6 Alpha acceptability test

Alpha acceptability testing of the DA prototype was conducted among stakeholders, including individuals with schizophrenia and health‐care providers. The test consisted of DA assessments in terms of length, content, balanced information presentation, and decision‐making ability.^27^ This is a common process of DA development using stakeholders' feedback to finalize the DA. The committee developed a mixed‐method survey according to the validated DA acceptability assessment questionnaires.^27^

Individuals with schizophrenia who were receiving antipsychotic treatment and health‐care providers who were routinely caring for those individuals were asked to review the prototype and complete the survey. Twenty individuals were approached from each group. The sample size was determined according to previous DA development studies, in which participants' acceptability was examined.^28^, ^29^ Results of acceptability test were then used to revise and improve the contents of the DA that would be suitable for use in actual clinical settings.

### 2.7 Assessment of the developed DA based on the IPDAS criteria

Finally, the developed DA was assessed using the IPDAS.^21^

Beta testing to examine its effectiveness was not performed because that was not the aim of the current study. The Ethics Board of Kyorin University, Tokyo Women's Medical University, and Shin‐Abuyama Hospital approved the study protocol. Participants in the alpha acceptability test were recruited from the Tokyo Women's Medical University and Shin‐Abuyama Hospital.

## 3 RESULTS

### 3.1 Development of the DA prototype

**FIGURE 2:**
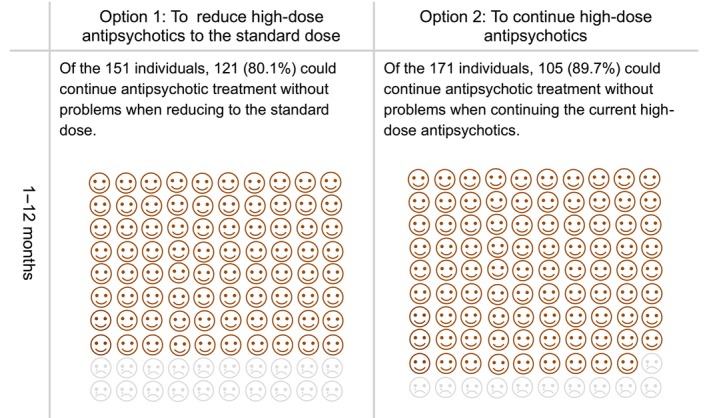
Pictorial diagrams showing the proportion of individuals who could continue antipsychotic treatment without any problem following the options to continue high‐dose antipsychotics or reduce to the standard dose.

**FIGURE 3:**
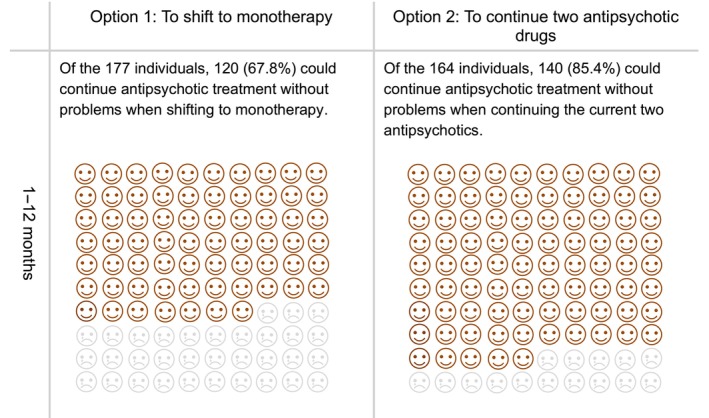
Pictorial diagrams showing the proportion of individuals who could continue antipsychotic treatment without any problems following the options to continue the current two antipsychotics or shift to monotherapy.

The prototype comprised a 32‐page A5 paper booklet. It contained an explanation of the target population of the DA, including ineligible conditions, how to use the DA, and a description of the symptoms of schizophrenia. The DA prototype provided options for antipsychotic monotherapy (to continue high‐dose antipsychotics or reduce to the standard dose), advantages and disadvantages of each option, and a value‐clarification worksheet for each option. Regarding the outcomes of the options, results of an additional subgroup analysis of previously conducted meta‐analysis were referred.^13^ In the group that reduced high‐dose antipsychotics to the standard dose, 30 out of 151 (19.9%) discontinued study participation due to all causes. In comparison, 12 out of 117 (10.3%) in the group that continued with high‐dose antipsychotics discontinued study participation due to all causes.^13^ The subgroup analysis showed no significant differences in study discontinuation due to all causes between the two groups: to continue high‐dose antipsychotics and reduce to the standard dose (*N* = 5, *n* = 268, RR = 1.59, 95% CI = 0.85–2.94, *p* = 0.14).^13^ In addition, the DA described options for combined treatment of two antipsychotic drugs: to continue polypharmacy or shift to monotherapy, the pros and cons of the options, and a value‐clarification worksheet for each option. Regarding the outcomes of the options, the results of a previous meta‐analysis were cited.^12^ In the group shifting to monotherapy, 57 out of 177 (32.2%) discontinued study participation due to all causes, whereas 24 out of 154 (14.6%) discontinued study participation due to all causes in the group of continuing polypharmacy.^12^ This meta‐analysis found that study discontinuation due to all causes was lower in the option to continue polypharmacy (two antipsychotic drugs) than in the option to shift to monotherapy (*N* = 6, *n* = 341, RR = 2.28, 95% CI = 1.50–3.46, *p* = 0.0001).^12^ To describe these outcomes for each option, pictorial diagrams of 100 faces were used, where shaded faces represented the proportion of individuals predicted to experience each outcome (Figures 2 and 3).

In the Appendices of the DA prototype, useful information such as chlorpromazine‐equivalent doses for antipsychotics, side effects of antipsychotics, concept of recovery, and coping strategies to promote recovery were provided. Appendix S1 presents the content and rationale of the DA prototype.

### 3.2 Alpha acceptability test

#### 3.2.1 Patients

Twenty individuals with schizophrenia who were undergoing antipsychotic treatment reviewed the DA prototype and completed the survey. The mean age of the participants was 47.7 years, and 12 (60%) were women. Table 1 shows the results of the four‐point Likert scale used to assess how well the information is described in each section of the DA prototype. The length of sentences was considered appropriate in 15 of the 20 (75%) participants; the amount of information was considered appropriate in 15 of the 20 (75%) participants; 15 of the 19 (79%) participants reported that the presentation was well‐balanced; 15 of the 18 (83%) participants believed that the DA was useful for the decision whether to reduce antipsychotics; 13 of the 19 (68%) participants assessed that they could foresee the outcomes of the two options in the DA prototype; 13 of the 17 (76%) participants considered that the DA made decision‐making easy; and 17 of the 19 (89%) participants reported that the DA had sufficient information to help them decide whether to reduce antipsychotics.

**TABLE 1:** Patient assessment of how information was presented in each section of the prototype.

	Mean	SD
About this booklet (*n* = 20)	2.75	0.55
What is schizophrenia? (*n* = 20)	2.74	0.87
Monotherapy		
Further treatment options (*n* = 20)	2.60	0.75
Comparing the pros and cons of each option (*n* = 20)	2.60	0.68
Comparing the consequences of each option (*n* = 10)	2.90	0.32
Value clarification (*n* = 20)	2.80	0.77
Preparation for SDM (*n* = 20)	2.65	0.81
Combined use of two antipsychotic drugs		
Further treatment options (*n* = 19)	2.63	0.76
Comparing the pros and cons of each option (*n* = 19)	2.68	0.67
Comparing the consequences of each option (*n* = 19)	2.79	0.42
Value clarification (*n* = 19)	2.79	0.85
Preparation for shared decision‐making (*n* = 19)	2.63	0.83
Appendices (*n* = 19)	2.89	0.66

*Note*: Rating system: four‐point Likert scale ranging from 1 to 4, with 4 = excellent, 3 = good, 2 = fair, and 1 = poor.

Abbreviation: SD, Standard deviation.

Narrative feedback on the DA prototype was relatively positive. Some comments are provided below.

“I found the diagrams and tables easier to understand than the text alone.”

“I liked the policy of deciding together.”

“It contained information that I found quite difficult to ask my doctor during consultation.”

“Both pros and cons of each option were provided, which was helpful.”

“Even 1% is important to me.”

“This helped me know that there were many types of drugs.”

“The Appendix, particularly the examples of strategies for recovery, was useful.”

“The symptoms vary from person to person; thus, it is difficult to apply the information to everyone, but the information on treatment will be useful for everyone.”

However, some participants complained that the DA lacked specific examples and experiences of others who were in similar situations.

#### 3.2.2 Health‐care providers

A total of 20 health‐care providers, including psychiatrists, nurses, pharmacologists, and psychologists, completed the questionnaire. The mean age was 34.6 years; six were women, five were men, and one was unknown. Overall, the perceptions of the DA prototype were favorable (Table 2).

**TABLE 2:** Health‐care providers' perceptions of the DA prototype (*n* = 20).

	Mean	SD
It will be easy for me to use	3.95	0.69
It is easy for me to understand	3.55	0.76
It will be easy for me to experiment with using the strategy before making a final decision to adopt it	3.65	0.67
The results of using the strategy will be easy to see	4.15	0.67
This strategy is better than how I usually go about helping patients decide whether to reduce antipsychotics	3.85	0.88
This strategy is compatible with the way I think things should be done	3.95	0.69
The use of this strategy is more cost‐effective than my usual approach to helping patients decide whether to reduce antipsychotics	3.60	0.68
Compared with my usual approach, this strategy will help my patients make more informed decisions	4.20	0.70
Using this strategy will save me time	2.85	0.81
This strategy is a reliable method for helping patients decide whether to reduce antipsychotics	4.15	0.81
Parts or components of the strategy can be used personally	3.80	0.70
This strategy is suitable for helping patients make value‐laden choices	4.15	0.59
This strategy complements my usual approach	3.75	0.64
Using this strategy does not involve making major changes to the way I usually do things	3.50	0.83
There is a high probability that using this strategy may cause/result in more benefit than harm	4.15	0.75

*Note*: Scores ranged from 1 (strongly disagree) to 5 (strongly agree).

Abbreviation: SD, Standard deviation.

Health‐care providers' positive feedbacks were described below:

“The information seems to be understandable for patients and their families, as the text is large and generally easy to read.”

“I like that the patient can write their own ideas in the booklet.”

“We can follow the steps, which can proceed decision‐making.”

“The information in the Appendix allows patients to check whether the amount of medication they are receiving is correct.”

“Using this booklet, health‐care providers arrive at a common perspective regarding antipsychotic medication treatment without personal bias.”

Furthermore, several suggestions were also provided:

“The sentence ‘Continuation of antipsychotic medication treatment is important to prevent relapse’ should be emphasized in bold.”

“Terms related to side effects, such as extrapyramidal symptoms and autonomic nervous system, should have furigana (way of pronunciation) as well.”

“Doctors who use the information in the booklet need to be properly familiar with how to use the booklet beforehand.”

“For each type of decision‐making, it may be useful to prepare two types of DA, which would be simple and easy.”

### 3.3 DA prototype modification based on stakeholders' feedback

The DA steering committee members assembled and discussed the acceptability test results. The responses and suggestions used to modify the DA prototype were examined. Regarding feedback from patients who desired examples of others, discussions whether to include personal stories in addition to the results of the meta‐analysis were considered. Consequently, personal experiences in the DA, considering that they were not based on evidence and might have led to a biased presentation of information, were excluded. In response to suggestions from health‐care providers that doctors should be familiar with using the booklet, a manual for them using DA was developed in Japanese and English (Appendix S2).

### 3.4 Development of the final DA

**FIGURE 4:**
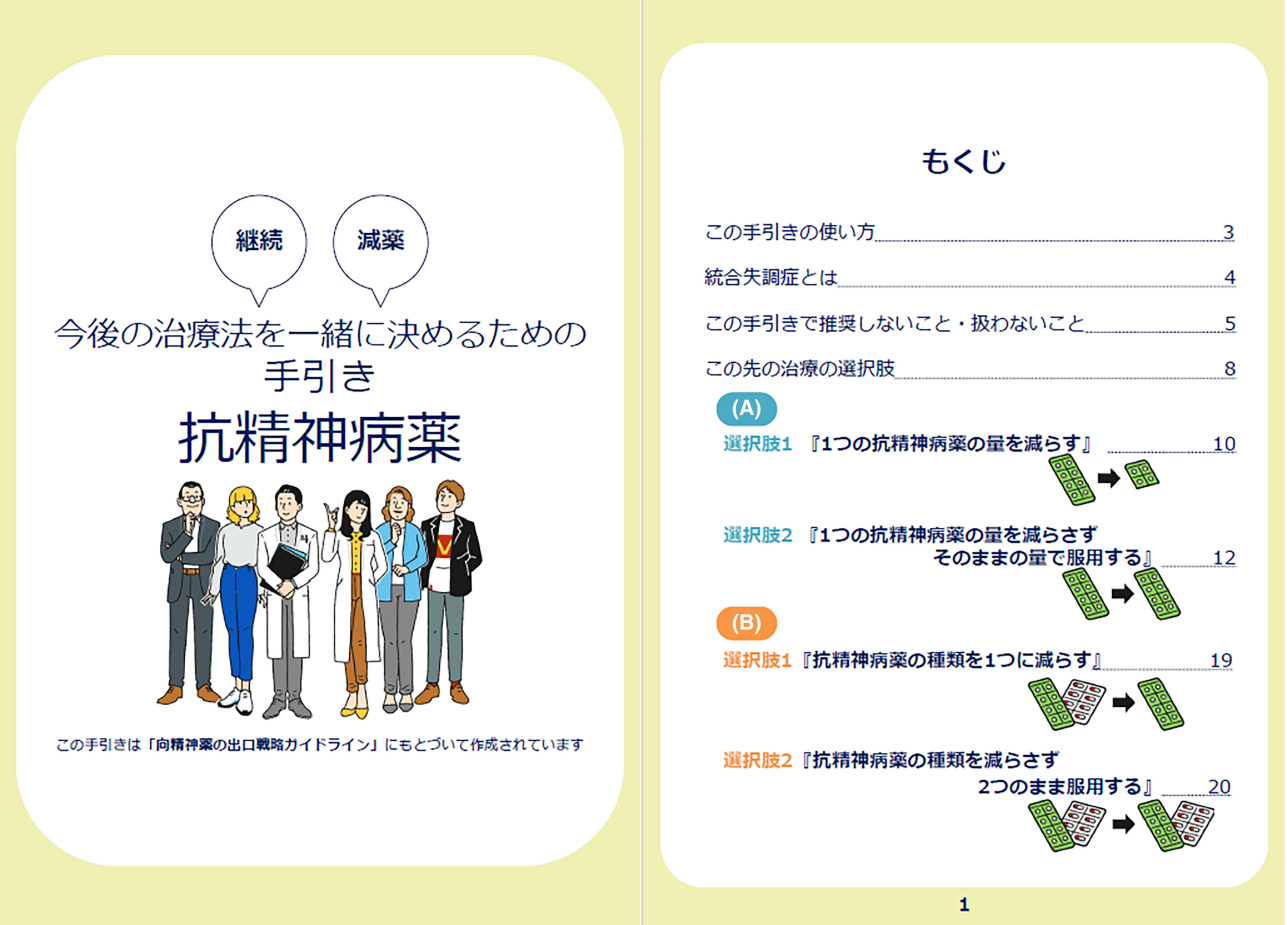
Cover page and Table of Contents described in the developed decision aid.

**FIGURE 5:**
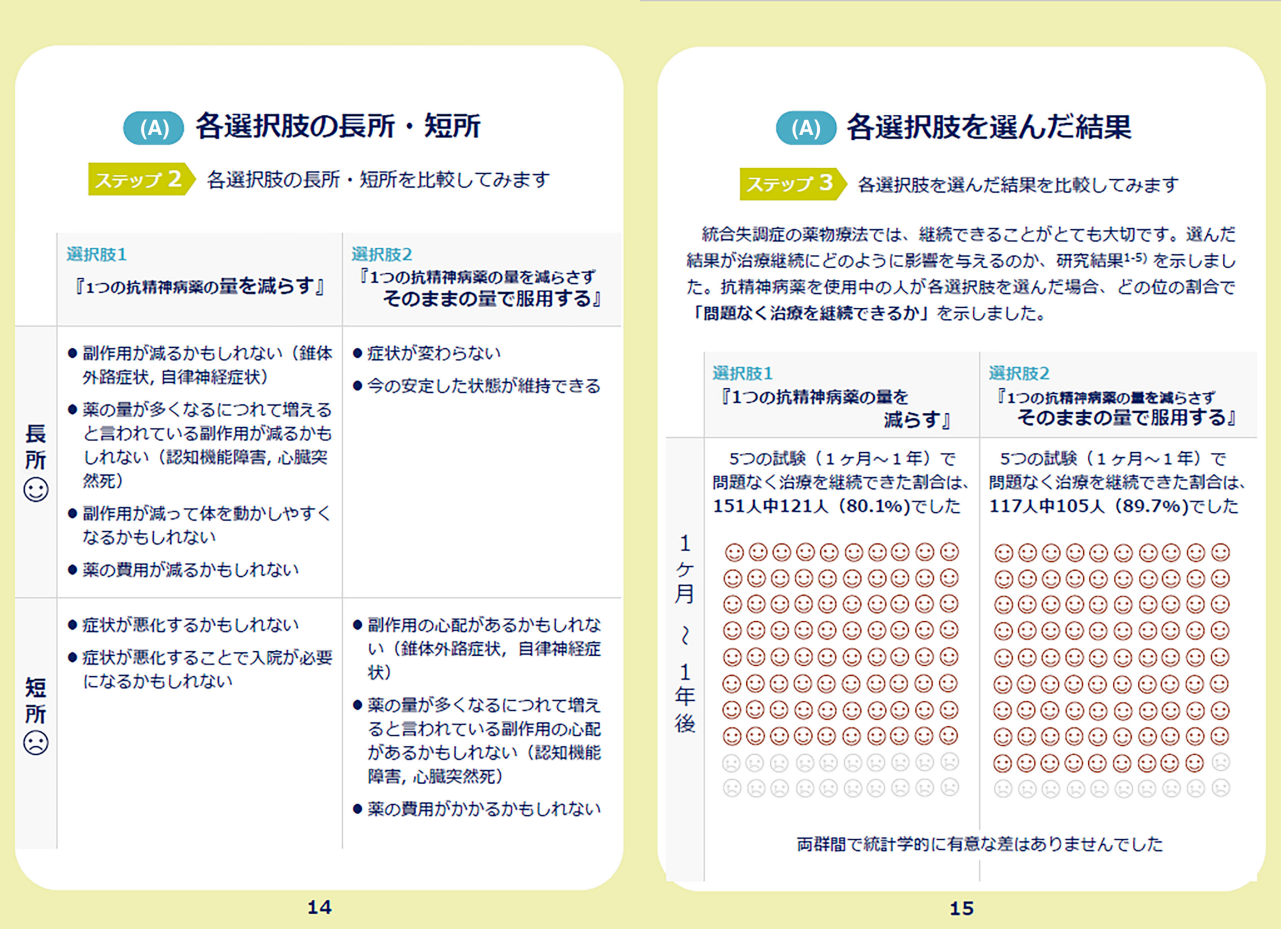
Comparison of the pros and cons of each option (left); comparison of the consequences of each option (right) for antipsychotic monotherapy in the developed decision aid.

**FIGURE 6:**
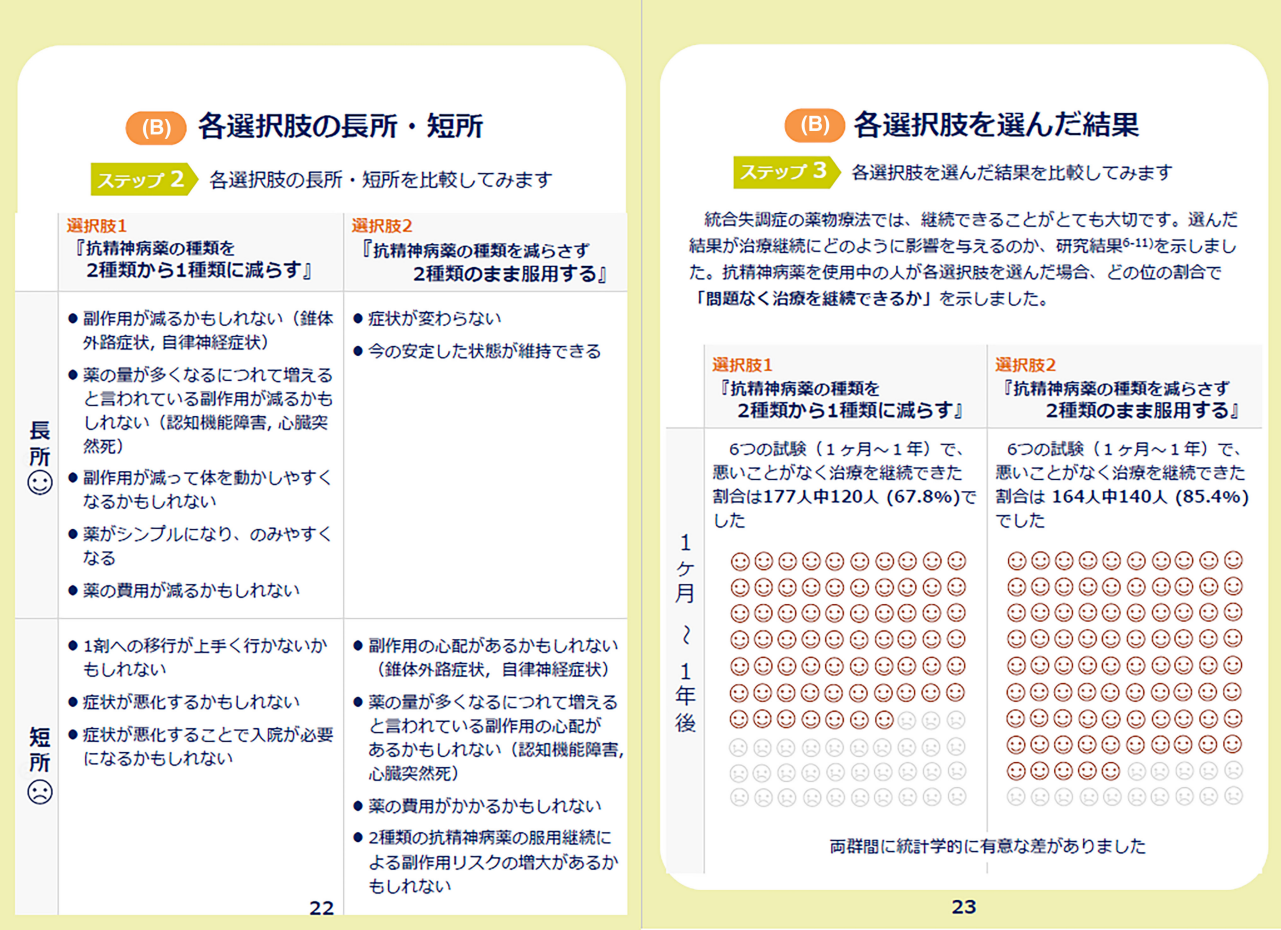
Comparison of the pros and cons of each option (left); comparison of the consequences of each option (right) for the combined use of antipsychotic drugs in the developed decision aid.

The final DA, which is available in both Japanese and English (Appendix S3), attained a high‐quality as determined by international DA criteria (Appendix S4). Our DA covered all IPDAS qualifying criteria (six of six), which was essential to be considered a DA. Our DA also met all IPDAS certification criteria (six of six). In cases where all of the certification criteria are not met, the DA is considered to have a high risk of harmful bias.^21^ The DA met most of the IPDAS‐quality criteria (18 of 23), which are deemed to enhance a DA but their absence does not mean a high risk of harmful bias.^21^ It has been found that the final DA met a highly rated criteria of the IPDAS, similar to other DAs accessible on the Ottawa DA website^30^ that address various health issues or health‐care screenings. (Figures 4, 5 and 6).

## 4 DISCUSSION

This is the first study on the development and acceptability testing of DA for individuals with stable schizophrenia, considering the reduction in the current antipsychotic treatment. The results suggest that the developed DA, a conversation and treatment decision‐support tool, was acceptable to stakeholders, particularly individuals with schizophrenia and health‐care providers.

Patient participation has gained attention in the mental health field^15^ similar to other somatic areas.^16^ In addition, the majority of individuals with schizophrenia desire to participate in their own treatment decisions.^31^ Several trials have been conducted to investigate the effects of patient involvement in schizophrenia treatment. For example, a comprehensive outpatient system developed by Yamaguchi et al.^32^ consisting of peer support and computerized decision‐support tools, appeared to improve patients' perceptions of communication and relationships with doctors. The trend of adopting a patient‐centered approach is also seen in acute psychiatric wards. Ishii et al. suggested that conversations between patients and interprofessional teams about ongoing treatments were feasible even upon the first admission for schizophrenia.^33^ Hamann et al. reported that individuals with schizophrenia receiving training of treatment participation exhibited better therapeutic alliance and satisfaction during acute hospital stay.^34^ Our DA may be useful as a conversation tool between patients and health‐care providers in both outpatient and inpatient care and can contribute to the existing literature on the patient participation in psychiatric treatment.

The strength of this study lies in the analysis of antipsychotic polypharmacy. Although continued antipsychotic treatment is essential for preventing relapse, antipsychotic polypharmacy has been associated with adverse effects,^6^, ^7^, ^8^, ^9^, ^10^ which can cause nonadherence to prescribed medication.^35^ Therefore, a strategy of shifting from antipsychotic polypharmacy to antipsychotic monotherapy was emphasized in clinical settings.^36^ However, there are little interventions for polypharmacy/high‐dose antipsychotic management that include patient participation during the drug/dose reduction process. Even among individuals who experienced adverse effects caused by antipsychotic polypharmacy and strongly requested antipsychotic drug/dose reductions, many patients were anxious about these reductions.^36^ Therefore, it is important that health‐care providers advocate patients' perspectives and opinions during the drug reduction process. Our DA can play the role of promoting a two‐way conversation in the drug reduction process. Nguyen et al.^37^ suggested that there were communication failures whereby de‐prescribing plans were not implemented and patients' misinformed views translated to medication‐seeking behavior as the external factors of antipsychotic polypharmacy. Ours will be used to share drug management plans to bridge communication gaps and live up to patients' medication expectations with information provision, that may improve antipsychotic medication adherence.

Although some participants preferred that examples and personal stories were added to the DA, the committee members discussed and decided not to provide subjective information on the final DA. Including personal stories has both strengths and weaknesses. Personal stories can support individuals who have to take health‐care decisions based on their own preferences by helping them explore their values^38^, ^39^; however, they have the potential to provide a biased view of those who experienced the situation.^40^ Adequate evidence has not been provided for personal stories on the DA. Therefore, like most other published DAs that do not have personal stories,^30^ ours focused on evidence‐based outcomes as information related to the options.

This study has some limitations. Our DA met most of the IPDAS‐quality criteria^21^; however, several elements, such as field testing and evidence, should be met in the future. Accordingly, field beta testing among stakeholders should be conducted. Therefore, it is necessary to examine the intervention effects of the developed DA during the SDM process in clinical settings. Moreover, it may be challenging for individuals with insufficient disease insight to read or utilize this decision aid independently. In such situations, using DA through communication with caregivers may be helpful. It is important to promote shared decision‐making involving caregivers, including family members and health‐care providers, while utilizing DA.

In conclusion, A DA was developed for individuals with stable schizophrenia, considering whether to reduce antipsychotics. The DA was acceptable for both patients and health‐care providers. As a next step, a field beta testing should be conducted to examine the intervention effects of the DA during the SDM process in clinical settings.

## AUTHOR CONTRIBUTIONS

YA involved in study design, drafting and revision of the DA prototype, data analysis and interpretation, revision of the DA, and manuscript drafting. Y Takaesu was the corresponding author and study design, revision of the DA prototype, data analysis and interpretation, revision of the DA, and manuscript editing. K Matsui, TT, H Tani, Y Takekita, T Kanazawa, T Kishimoto, ST, NH, and H Takeuchi involved in study design, revision of the DA prototype, data interpretation, revision of the DA, and manuscript editing. KI involved in study design, revision of the DA prototype, data collection and interpretation, revision of the DA, and manuscript editing. K Mishima involved in study design, revision of the DA prototype, data interpretation, revision of the DA, manuscript editing, and funding acquisition. All authors made substantial contributions to the conception and design, acquisition of data, or analysis and interpretation of data; took part in drafting or revising the manuscript for important intellectual content; agreed to submit the manuscript to the current journal; gave final approval of the version to be published; and agreed to be accountable for all aspects of the study.

## FUNDING INFORMATION

This study was supported by research grants from the Ministry of Health, Labor, and Welfare of Japan (21GC1016) and Grants‐in‐Aid for Scientific Research (20K10792).

## CONFLICT OF INTEREST STATEMENT

Yumi Aoki has received speaker's honoraria from Sumitomo Pharma, Meiji Seika Pharma, Viatris Pharmaceuticals Japan. Yoshikazu Takaesu has received lecture fees from Takeda Pharmaceutical, Sumitomo Dainippon Pharma, Otsuka Pharmaceutical, Meiji Seika Pharma, Kyowa Pharmaceutical, Eisai, MSD, Yoshitomi, and research funding from Otsuka Pharmaceutical, Meiji Seika Pharma, MSD, and Eisai. Kentaro Matsui reports personal fees from Eisai, Meiji Seika Pharma, MSD, Otsuka Pharmaceutical, Takeda Pharmaceutical, and Yoshitomi Pharmaceutical, outside the submitted work. Takahiro Tokumasu reports personal fees from Meiji Seika Pharma, Sumitomo Pharmaceutical, Jansen Pharmaceutical, Otsuka Pharmaceutical and Takeda Pharmaceutical outside the submitted work. Hideaki Tani has received manuscript or speaker fees from Sumitomo Pharma, Janssen Pharmaceutical, Otsuka Pharmaceutical, Takeda, Wiley Japan, and Yoshitomi Yakuhin within the past 3 years. Yoshiteru Takekita has received grant funding from the Japan Society for the Promotion of Science and speaker's honoraria from Meiji‐Seika Pharma, Sumitomo Pharma, Janssen Pharmaceutical, Otsuka, Eisai, Daiichi‐Sankyo, Pfizer, UCB Japan, Takeda Pharmaceutical, Lundbeck Japan KK, Novartis and Teijin Pharma. Tetsufumi Kanazawa received personal fees as speaker's honoraria from Eisai, Janssen, Kyowa, Meiji Seika Pharma, MSD, Otsuka, Pfizer, Shionogi, Sumitomo Pharma, Takeda, Yoshitomiyakuhin, Viatris, and he received research grant support from Eisai, Ostuka, and Sumitomo pharma in the last 3 years. Taishiro Kishimoto reports consultant fees from Boehringer‐Ingelheim, Chugai, Otsuka, Sumitomo Pharma, speaker's honoraria from Eisai, Janssen, Mitsubishi Tanabe, Mochida, MSD, Novartis, Otsuka, Pfizer, Sumitomo Pharma, license fee from Sumitomo Pharma, and receiving donation course from Mori building, outside the submitted work. Seiichiro Tarutani has received honoraria for lectures from Otsuka Pharmaceutical Co., Ltd., Sumitomo Dainippon Pharma Co., Ltd., Meiji Seika Pharma Co., Ltd., Janssen Pharmaceutical K.K., and Yoshitomiyakuhin Co., Naoki Hashimoto has received consultant fees from Janssen Pharma, Sumitomo Pharma, speaker's honoraria from Janssen, Otsuka, Sumitomo Pharma, Janssen Pharma, Yoshitomi Yakuhin, Novartis Pharma, Meiji‐Seika Pharma, Takeda Pharmaceutical, Hiroyoshi Takeuchi has received grants from Daiichi Sankyo and Novartis Pharma; speaker's fees from EA Pharma, Eisai, Kyowa, Janssen, Lundbeck, Meiji Seika Pharma, MSD, Otsuka, Sumitomo Pharma, Takeda, and Yoshitomiyakuhin; and consulting fees from Janssen, Mitsubishi Tanabe Pharma, Ono, and Sumitomo Pharma. Kazuo Mishima has research grants from Eisai Co., Ltd., Sumitomo Pharma Co., Ltd., Takeda Pharmaceutical Co., Ltd., SONY Corporation and also received speaker's honoraria from Eisai Co., Ltd., Nobelpharma Co., Ltd., Takeda Pharmaceutical Co., Ltd., MSD Inc., Otsuka. Pharmaceutical Co., Ltd., Viatris Inc., outside the submitted work. Ken Inada received personal fees from Daiichi‐Sankyo, Eisai, Eli Lilly, Janssen, Lundbeck Japan, Meiji Seika Pharma, Mitsubishi Tanabe Pharma, Mochida, MSD, Nipro, Novartis, Otsuka, Pfizer, Shionogi, Sumitomo Pharma, Yoshitomiyakuhin, Viatris, and he received research grant support from Mochida and Sumitomo pharma in the last 3 years.

## ETHICAL APPROVAL

Approval of the research protocol by an Institutional Reviewer Board: The Ethics Board of Kyorin University approved the study protocol.

Informed consent: Written informed consent was obtained from the participants.

Registry and the Registration No. of the study.

Trial (if not applicable please write n/a).

Animal studies: n/a.

## Supporting information


Appendix S1

Appendix S2

Appendix S3

Appendix S4

Appendix S5

Appendix S6

Appendix S7

## Data Availability

The data supporting the results reported in the article is available in the supplementary information.
