# Supplementary material for: Development and acceptability testing of a decision aid for considering whether to reduce antipsychotics in individuals with stable schizophrenia
Source: Neuropsychopharmacol Rep. 2023 Jul 14;43(3):391–402. doi: 10.1002/npr2.12366 (PMC10496039; doi:10.1002/npr2.12366)
Supplement: Supplementary file 1 — Appendix S1 [file NPR2-43-391-s007.pdf]

Data supporting the results reported in the article

Patients

	1	2	3	4	5	6	7	8	9	10	11	12	13	14	15	16	17	18	19	20
About this booklet	3	3	3	2	3	2	2	2	3	3	3	3	3	4	2	3	3	2	3	3
What is schizophrenia?	3	3	3	.	3	2	2	1	3	3	4	3	3	4	3	4	3	2	2	1
Monotherapy:																				
Further treatment options	2	3	3	2	2	2	2	4	3	2	3	3	3	4	3	3	3	2	2	1
Comparing the pros and cons of each option	3	3	3	2	2	2	3	3	3	2	2	3	4	3	3	3	2	2	3	1
Comparing the consequences of each option	.	.	.	.	.	.	.	.	.	.	3	3	3	3	3	3	3	3	2	3
Value clarification	2	3	3	2	4	3	3	3	3	2	3	3	3	4	3	3	4	2	2	1
Combined use of two antipsychotic drugs:																				
Preparation for SDM	2	3	3	1	2	3	3	3	3	2	3	3	3	4	3	3	4	2	2	1
Further treatment options	3	3	3	1	3	3	2	3	3	2	4	3	3	3	.	3	3	2	2	1
Comparing the pros and cons of each option	3	3	3	1	3	3	2	3	3	2	4	3	3	3	.	3	2	2	2	3
Comparing the consequences of each option	3	3	3	2	3	3	2	3	3	3	3	3	3	3	.	3	3	3	2	2
Value clarification	3	3	3	1	3	3	2	3	3	3	4	3	3	4	.	3	4	2	2	1
Preparation for shared decision making	3	3	3	1	3	3	2	3	3	2	2	3	3	4	.	3	4	2	2	1
Appendices	2	3	3	2	3	3	2	3	3	2	3	3	4	3	.	4	4	3	2	3

Health-care providers

	1	2	3	4	5	6	7	8	9	10	11	12	13	14	15	16	17	18	19	20
It will be easy for me to use.	3	4	3	4	3	4	4	4	5	3	3	4	4	4	4	5	4	4	5	5
It is easy for me to understand.	3	4	3	3	3	4	3	4	4	2	2	4	4	4	3	5	4	4	4	4
It will be easy for me to experiment with using the strategy before making a final decision to adopt it.	4	4	5	3	3	3	4	3	4	3	3	3	4	3	3	4	4	4	5	4
The results of using the strategy will be easy to see.	4	4	5	3	4	3	4	3	5	4	5	4	4	4	4	5	4	5	5	4
This strategy is better than how I usually go about helping patients decide whether to reduce antipsychotics.	3	4	5	3	3	3	4	3	5	3	3	3	5	3	5	5	4	4	5	4
This strategy is compatible with the way I think things should be done.	4	4	5	4	3	3	4	3	3	5	3	4	4	4	4	5	4	5	4	4
The use of this strategy is more cost-effective than my usual approach to helping patients decide whether to reduce antipsychotics.	3	4	3	3	3	3	4	4	3	4	4	4	3	4	5	5	3	4	3	3
Compared with my usual approach, this strategy will help my patients make more informed decisions.	4	4	5	4	4	3	5	3	4	4	5	5	3	4	4	5	5	4	4	5
Using this strategy will save me time.	4	4	2	2	3	3	3	2	2	2	2	3	2	4	3	4	3	3	2	4
This strategy is a reliable method for helping patients decide whether to reduce antipsychotics.	4	4	5	4	4	4	5	3	4	4	5	3	2	4	5	5	4	4	5	5
Parts or components of the strategy can be used personally.	4	4	3	2	4	3	4	3	4	4	4	4	3	4	4	4	4	4	5	5
This strategy is suitable for helping patients make value-laden choices.	3	4	5	4	4	3	4	4	5	4	4	4	4	4	4	5	4	4	5	5
This strategy complements my usual	3	4	4	3	4	3	4	3	3	4	4	4	3	4	4	5	4	4	5	3
Using this strategy does not involve making major changes to the way I usually do things.	3	4	5	4	4	2	2	3	3	4	4	3	3	3	3	4	4	5	3	4
There is a high probability that using this strategy may cause/result in more benefit than harm.	3	4	4	3	4	4	5	4	5	5	3	5	3	4	4	5	4	5	5	4
