# Supplementary material for: Development and acceptability testing of a decision aid for considering whether to reduce antipsychotics in individuals with stable schizophrenia
Source: Neuropsychopharmacol Rep. 2023 Jul 14;43(3):391–402. doi: 10.1002/npr2.12366 (PMC10496039; doi:10.1002/npr2.12366)
Supplement: Supplementary file 2 — Appendix S2 [file NPR2-43-391-s004.pdf]

Appendix 1. Content and rationale of the DA

Content	Pages	*Rationale*/Reference
**About this booklet**		
Description of the decision to be considered<br>Explanation of the target population<br>Instructions on the use of the booklet	1–3	*IPDASi Qualifying Criteria: The patient decision aid explicitly states the decision that needs to be considered (index decision).*
**What is schizophrenia?**		
Objective information about schizophrenia, such as symptoms, recovery as a goal, treatment, and course<br>Explanation for non-recommendations on this DA, including discontinuation of medication	3–7	*IPDASi Qualifying Criteria: The patient decision aid describes the health condition or problem for which the index decision is required.*<br>- Leamy et al. Br J Psychiatry. 199: 445–452, 2011.<br>- Gardener et al. Am J Psychiatry. 167: 686–693, 2010.
**A: Monotherapy**		
**Step 1: Further treatment options**		
Options provided:<br>Option 1: To continue the current high-dose antipsychotics<br>Option 2: To reduce to the standard dose	9–13	*IPDASi Qualifying Criteria: The patient decision aid describes the options available for the index decision.*
**Step 2: Comparing the pros and cons of each option**		
A table comparing the options (advantages and disadvantages)	14	*IPDASi Qualifying Criteria: The patient decision aid describes the positive features of each option.*<br>*IPDASi Qualifying Criteria: The patient decision aid describes the negative features of each option.*<br>- Gardener et al. Am J Psychiatry. 167: 686–693, 2010.<br>- Watanabe. Nervous system agents in Current Drug Therapy, NANKO-DO, 2021 (in Japanese).
**Step 3: Comparing the consequences of each option**		
Pictorial diagrams of the recurrence rates for each option	15	*IPDASi Qualifying Criteria: The patient decision aid describes what it is like to experience the consequences of the options.*<br>-Tani, et al. Neuropsychopharmacology. 45:887–901, 2020.
**Step 4: Value clarification**		
A value-clarification exercise with a 5-point Likert scale	16	*IPDASi Qualifying Criteria: The patient decision aid asks patients to identify which positive and negative features of the options are important to them.*
**Step 5: Preparation for shared decision making**		
Memo field to prepare for decision-making consultation	17	I*PDASi Qualifying Criteria: The patient decision aid includes tools such as worksheets or lists of questions to use when discussing options with a practitioner.*
**A: Combined use of two antipsychotic drugs**		
**Step 1: Further treatment options**		
Options provided:<br>Option 1: To continue the current two antipsychotics.<br>Option 2: To shift to monotherapy	18–21	*IPDASi Qualifying Criteria: The patient decision aid describes the options available for the index decision.*

Appendix 1. Continued

**Step 2: Comparing advantages and disadvantages of each option**		
A table comparing options (advantages and disadvantages)	22	*IPDASi Qualifying Criteria: The patient decision aid describes the positive features of each option.*<br>*IPDASi Qualifying Criteria: The patient decision aid describes the negative features of each option.*<br>- Gardener et al. Am J Psychiatry. 167: 686–693, 2010.<br>- Watanabe. Nervous system agents in Current Drug Therapy, NANKO-DO, 2021 (in Japanese)
**Step 3: Comparing the consequences of each option**		
Pictorial diagrams of the recurrence rates for each option	23	*IPDASi Qualifying Criteria: The patient decision aid describes what it is like to experience the consequences of the options.*<br>-Matsui, et al. Schizophr Res. 209:50–57, 2019.
**Step 4: Value clarification**		
A value-clarification exercise with a 5-point Likert scale	24	*IPDASi Qualifying Criteria: The patient decision aid asks patients to identify which positive and negative features of the options are important to them.*
**Step 5: Preparation for shared decision making**		
Memo field to prepare for decision-making consultation	25	I*PDASi Qualifying Criteria: The patient decision aid includes tools such as worksheets or lists of questions to use when discussing options with a practitioner.*
**Appendices**		
- Chlorpromazine-based dose<br>- Side effects of antipsychotics<br>- Recovery<br>- Coping strategies to promote recovery<br>- Details of side effects of antipsychotics	26-32	Chlorpromazine-based dose:<br>-Inagaki and Inada. Japanese Journal of Clinical Pharmacology. 18:1457–1480, 2015 (in Japanese).<br>-Inagaki and Inada. Japanese Journal of Clinical Pharmacology. 20:89–97, 2017 (in Japanese).<br>Side effects:<br>-Watanabe. Nervous system agents in Current Drug Therapy, NANKO-DO, 2021 (in Japanese).<br>Recovery/Coping strategies to promote recovery.<br>-Yamaguhi and Kumakura. Journal of Clinical and Experimental Medicine (IGAKU NO AYUMI). 261: 941-948, 2017 (in Japanese).<br>-Leamy et al. Br J Psychiatry. 199-:445–452, 2011.<br>Details of side effects of antipsychotics<br>-Uchida et al. transl. Talor et al. The Maudsley Prescribing Guidelines in Psychiatry 13th Edition, Wiley Publishing Japan, 2019 (in Japanese).

IPDASi, International Patient Decision Aid Standards instrument; CBT, cognitive behavioral therapy
