# Supplementary material for: Development and acceptability testing of a decision aid for considering whether to reduce antipsychotics in individuals with stable schizophrenia
Source: Neuropsychopharmacol Rep. 2023 Jul 14;43(3):391–402. doi: 10.1002/npr2.12366 (PMC10496039; doi:10.1002/npr2.12366)
Supplement: Supplementary file 3 — Appendix S3 [file NPR2-43-391-s001.pdf]

継続/減薬：今後の治療法を一緒決めるための手引き － 抗精神病薬

― 医療者マニュアル ―

この手引きについて/手引きの使い方

【この手引き（DA）について】

この「継続/減薬：今後の治療法を一緒に決めるための手引き（Decision Aid, DA）-抗精神病薬」は、患者と一緒に共同意思決定（shared decision making, SDM)の手法で治療法を決める際に活用できるツールである。DA は、各選択肢の利点・欠点を提示し、本人の好みや価値観を含めた意向を確認しながら話し合い、患者と一緒に意思決定を行うための補助資材である。したがって、**患者にこれを渡して単独で決めてもらうものではなく、本資材を用いて医療者と患者での双方向性の話し合いの基に意思決定を行う**。

本 DA は、①「高用量の抗精神病薬が処方されている患者において、抗精神病薬を減量するか、あるいは継続するか」、および②「抗精神病薬が 2 剤併用されている患者において、抗精神病薬を単剤化するか、あるいは継続するか」について、SDM の手法で患者と一緒に話し合いながら決めるための補助資材である。

【この手引き（DA）の対象】

本 DA の対象は、診断が確定し、症状が安定している維持期の統合失調症患者における、高用量の抗精神病薬（クロルプロマジン換算 600mg/日以上）からの減量、または2剤から1剤への単剤化である。

抗精神病薬の休薬、断薬については、病状の悪化につながることを示す強いエビデンスがあり、推奨しない。症状が不安定な患者における減量、単剤化は、病状が悪化する可能性があり、推奨しない。

本 DA の単剤化の根拠となった研究は全て2剤の抗精神病薬を1剤に減らす研究であり、3剤以上の抗精神病薬の併用からの単剤化は、対象としない。抗精神病薬以外の向精神薬の併用については、本 DA の趣旨から外れており、対象としない。

なお、統合失調症治療における3剤以上の抗精神病薬の併用や、抗精神病薬以外の向精神薬の使用については、これを支持する強いエビデンスは存在しない。これらについて患者から減薬を相談された場合は、その処方とならざるを得なかった理由について十分に説明の上、減薬の可能性について議論し、共同意思決定に努めることが望ましい。

【この手引き（DA）の使い方】（P3）

はじめに本 DA の使い方について患者と共有する。すぐに結論を出すのではなく、この資材を活用しながら、一緒に決めていくことを強調する。以降のページでは、読み進めながら○をつけたり書き込んだりできるようになっていることを説明し、疑問や感想、書き込んだ内容について、次回話し合おうと伝える。

整理減量の 2 つのパターン：パターン A 総量の減量／パターン B 単剤化

パターン A 総量の減量

【ステップ1】選択肢の説明（P9 – P13）

抗精神病薬の減量とは、現在処方されている抗精神病薬の用量を減らすことである。本 DA では、クロルプロマジン換算 600mg/日以上の高用量の抗精神病薬が処方されている患者を対象としている。なお、等価換算は、付録 3 の Gardner らの論文(2010 年)[1] に基づいており、クロザピン導入時の基準として用いられているクロルプロマジン換算量とは一部異なる。

ここでは、「抗精神病薬の用量を減量する」、「抗精神病薬の用量を継続する」という 2 つの選択肢について患者に理解してもらう。いずれを選択した場合も抗精神病薬は継続して使用することが大切である点は十分に説明する。また、付録２で示されている「日常生活でリカバリーのためにできること」を開始・継続することが寛解（回復）を維持する上で重要である。

【ステップ2】長所と短所の確認（P14）

各選択肢の長所・短所を確認してもらう。付録 4・付録 5 も必要に応じて参照し、抗精神病薬の副作用の特徴を理解してもらう。

【ステップ 3】効果の比較（P15）

ピクトグラムを用いて各選択肢の効果（各選択肢を選んだ結果どうなるか）について理解してもらう。

このピクトグラムは、高用量の抗精神病薬（クロルプロマジン換算 600mg/日以上）からの用量減量と用量維持を比較した 5 つの無作為化対照試験のメタ解析 [2] の結果に基づき作成されている。主要転帰は「すべての理由による治療中断」であり、ピクトグラムは、中断に至らなかった患者の割合を選択肢ごとに示している。5 つの試験（試験期間：1 か月～1 年）において、治療の継続ができていたと推定される患者の割合は、用量減量群：121/151 人（80.1%）、用量維持群：105/117 人（89.7%）であり、両群の治療中断率に統計学的に有意な差は認められなかった（リスク比：1.59、95%信頼区間：0.85−2.94、p=0.14）。

ただし、抗精神病薬の減量の効果を検討した研究はまだ少数であり、今後さらなる研究が行われることで、統計学的な有意性は変わるかもしれない。また現時点では、1 年以下の比較的短期間の転帰についてのみエビデンスが得られている状況であることに注意が必要である。

【ステップ4】価値観（P16）

患者と話し合いながら、各選択肢に関する本人の価値観や好みを明らかにしていく。まず患者に、ステップ 2 で取り上げた各選択肢の長所・短所について、自身の重要度を評価してもらう。そして診察では、その数値（0~5）に〇をつけた理由について具体的に聞き取り、話し合う。つまり、単に高得点の多かった選択肢を自動的に選ぶのではなく、共同意思決定に向け、患者の価値観や好みといった主観的な情報を一緒に整理し、共有するためのステップである。その他、患者が自由記載した項目についても詳細を聞き、共同意思決定の材料にする。

【ステップ5】話し合いの準備（P17）

2 つの選択肢に関する疑問点、懸念事項を積極的に記入してもらう。ステップ 3、4 の記載内容に基づき、抗精神病薬を減量するか同じ量で継続するか話し合う。

パターン B 単剤化

【ステップ1】選択肢の説明（P18 – P21）

単剤化とは 2 剤以上の併用を 1 剤にすることである。

3 剤以上の併用を 2 剤にすることや 1 剤にすることも単剤化であるが、このような複雑な状況についての研究はほとんどなく、本 DA では扱っていないことに注意する。3 剤以上であれば、まず 2 剤に整理することを検討すべきである。

ここでは、「抗精神病薬 2 剤併用を単剤化する」、「抗精神病薬 2 剤併用を継続する」という 2 つの選択肢について患者に理解してもらう。いずれを選択した場合も抗精神病薬は継続して使用することが大切である点は十分に説明する。また、付録 2 で示されている「日常生活でリカバリーのためにできること」を開始・継続することが寛解（回復）を維持する上で重要である。

【ステップ 2】長所と短所の確認（P22）

各選択肢の長所・短所を確認してもらう。付録 4・付録 5 も必要に応じて参照し、抗精神病薬の副作用の特徴を理解してもらう。

【ステップ 3】効果の比較（P23）

ピクトグラムを用いて各選択肢の効果（各選択肢を選んだ結果どうなるか）について理解してもらう。

このピクトグラムは、抗精神病薬 2 剤からの単剤化と 2 剤併用の継続を比較した 6 つの無作為化対照試験のメタ解析[3]の結果に基づき作成されている。主要転帰は「すべての理由による治療中断」であり、ピクトグラムは、中断に至らなかった患者の割合を選択肢ごとに示している。6 つの試験（試験期間：1 か月～1 年）において、治療の継続ができていたと推定される患者の割合は、単剤化群：120/177 人（67.8%）、2 剤併用継続群：140/164 人（85.4%）であり、両群の治療中断率に統計学的に有意な差が認められた（リスク比：2.28、95%信頼区間：1.50－3.46、p=0.0001）。

ただし、抗精神病薬の単剤化の効果を検討した研究はまだ少数であり、今後さらなる研究が行われることで、統計学的な有意性は変わるかもしれない。また現時点では、1 年以下の比較的短期間の転帰についてのみエビデンスが得られている状況であることに注意が必要である。

【ステップ4】価値観（P24）

患者と話し合いながら、各選択肢に関する本人の価値観や好みを明らかにしていく。まず患者に、ステップ 2 で取り上げた各選択肢の長所・短所について、自身の重要度を評価してもらう。そして診察では、その数値（0~5）に〇をつけた理由について具体的に聞き取り、話し合う。つまり、単に高得点の多かった選択肢を自動的に選ぶのではなく、共同意思決定に向け、患者の価値観や好みといった主観的な情報を一緒に整理し、共有するためのステップである。その他、患者が自由記載した項目についても詳細を聞き、共同意思決定の材料にする。

【ステップ5】話し合いの準備（P25）

2 つの選択肢に関する疑問点、懸念事項を積極的に記入してもらう。ステップ 3、4 の記載内容に基づき、抗精神病薬を単剤化するか 2 剤のまま継続するか話し合う。

文献）

1. Gardner DM, Murphy AL, O'Donnell H, Centorrino F, Baldessarini RJ. International consensus study of antipsychotic dosing. *Am J Psychiatry*. 2010 Jun;167(6):686-93. doi: 10.1176/appi.ajp.2009.09060802.
2. Tani H, Takasu S, Uchida H, Suzuki T, Mimura M, Takeuchi H. Factors associated with successful antipsychotic dose reduction in schizophrenia: a systematic review of prospective clinical trials and meta-analysis of randomized controlled trials. *Neuropsychopharmacology*. 2020 Apr;45(5):887-901. doi: 10.1038/s41386-019-0573-7.
3. Matsui K, Tokumasu T, Takekita Y, Inada K, Kanazawa T, Kishimoto T, Takasu S, Tani H, Tarutani S, Hashimoto N, Yamada H, Yamanouchi Y, Takeuchi H. Switching to antipsychotic monotherapy vs. staying on antipsychotic polypharmacy in schizophrenia: A systematic review and meta-analysis. *Schizophr Res.* 2019 Jul;209:50-57. doi: 10.1016/j.schres.2019.05.030.
