# Supplementary material for: Development and acceptability testing of a decision aid for considering whether to reduce antipsychotics in individuals with stable schizophrenia
Source: Neuropsychopharmacol Rep. 2023 Jul 14;43(3):391–402. doi: 10.1002/npr2.12366 (PMC10496039; doi:10.1002/npr2.12366)
Supplement: Supplementary file 4 — Appendix S4 [file NPR2-43-391-s002.pdf]

A decision aid considering whether to reduce antipsychotics in individuals with stable schizophrenia
― A manual for healthcare providers ―

About this decision aid

【About this decision aid】

This decision aid (DA) can be used to help you and your patient decide on a treatment plan during shared decision making (SDM). SDM is a two-way decision-making process in which the patient and the healthcare provider decide together on the further treatment plan, which is a tool for making decisions together with the patient by sharing the advantages and disadvantages of each option, and discussing and confirming the patient's intentions, including preferences and values.

Therefore, <u>the DA is NOT intended to be handed over to the patient for him/her to decide independently.</u> The DA is a piece of equipment that helps decide (i) whether to continue high-dose antipsychotics or reduce to the standard dose and (ii) whether to continue two antipsychotics or shift to monotherapy, <u>through discussion with the patient during the SDM process</u>.

【Target of this DA】

The target of this DA is individuals with schizophrenia whose conditions are stable with undergoing antipsychotic treatment: (i) high-dose antipsychotic monotherapy (chlorpromazine-based dose ≥600 mg/day) or (ii) combined use of two antipsychotic drugs.

Withdrawal or abstinence from antipsychotics is not recommended because of strong evidence that it can worsen the disease state. Dose reduction and monotherapy in those with unstable symptoms is not recommended, as it may worsen the condition.

In all studies that provided the basis for monotherapy in this DA, two antipsychotics were reduced to one, and monotherapy from a combination of three or more antipsychotics was not covered. The concomitant use of psychotropics other than antipsychotics is outside the scope of this DA and not covered.

No strong evidence supports the concomitant use of three or more antipsychotics in schizophrenia treatment or the use of psychotropics other than antipsychotics. If patients inquire about tapering off these medications, it is desirable to discuss the reasons for prescribing them and explain thoroughly the possibility of tapering while striving for shared decision-making.

【How to use the DA】(P3)

First, share with the patient I how to use the DA. Emphasize that we will not come to a conclusion right away, but will make a decision together while using this tool. Explain that patients will be able to put a checkmark and write things down as they read, and tell patients that they can discuss their questions, impressions, and what they wrote down at the next visit.

Two patterns for reduction: pattern A, reduction in the total amount; pattern B, monotherapy

Patten A, reduction in total amount

【Step 1】Explanation of treatment options (P9 - P13)

Antipsychotic dose reduction refers to a reduction in the dose of an antipsychotic currently prescribed. In this DA, patients prescribed with high doses of antipsychotics with chlorpromazine equivalents of ≥600 mg/day are targeted. The equivalent conversions are based on the Gardner et al. (2010)[1] in Appendix 3, which differs in part from the chlorpromazine equivalent used as the standard for introducing clozapine.

Here, patients should be made aware of two options: reducing or continuing the dose of the antipsychotic. Whichever option is chosen, the importance of continued use of the antipsychotic should be fully explained. It is also important to initiate and continue the 'What you can do in your daily life to achieve recovery' goal as indicated in Appendix 2, in order to maintain remission (recovery).

【Step 2】Reviewing pros and cons of each treatment option (P14)

Patients will be asked to review the pros and cons of each option. Appendices 4 and 5 are also referred to as necessary to gain an understanding of the characteristics of the side effects of antipsychotics.

【Step 3】Comparing the consequences of each treatment option (P15)

A pictogram is used to help patients understand the effects of each option (what happens as a result of choosing each option).

It is based on the results of a meta-analysis[2] of five randomized controlled trials comparing dose reduction and dose maintenance from high-dose antipsychotics (>600 mg chlorpromazine equivalent/day). The primary outcome was treatment discontinuation due to all causes, and the pictogram shows the proportion of patients per option who did not discontinue across the five trials (study duration: 1 month to 1 year), the estimated proportion of patients who would have continued treatment was 121/151 (80.1%) in the dose reduction group and 105/117 (89.7%) in dose maintenance group, with no statistically significant difference in treatment discontinuation rates between the two groups (risk ratio: 1.59, 95% confidence interval: 0.85-2.94, $p = 0.14$).

However, only a few studies have examined the effect of antipsychotic dose reduction, and further studies may change the statistical significance. Currently, evidence is only available for relatively short-term outcomes of ≤1 year.

【Step 4】What is important to you? (Value clarification exercise) (P16)

In this section, we discuss with the patient to clarify his or her values and preferences regarding each option. First, the patient is asked to rate the importance of the pros and cons of each of the options discussed in Step 2. Then, during the consultation, the patient is specifically asked to explain why he or she marked the number 0–5. In other words, this is a

step to organize and share subjective information such as the patient's values and preferences together for shared decision-making, rather than simply automatically selecting the option with the highest score. Other items freely described by the patient should also be asked for details and used as material for shared decision-making.

【Step 5】Preparation for a consultation discussion (P17)

Ask the patient to proactively answer questions and concerns regarding the two options. Based on the information provided in steps 3 and 4, we discuss whether to continue or reduce antipsychotic medication.

Pattern B, monotherapy

【Step 1】Explanation of treatment options (P18 - P21)

Monotherapy is the reduction of a combination of two or more drugs to one drug.

Note that although reducing three or more concomitant drugs to two or one is also monotherapy, research on this complex situation is scarce and not addressed in this DA. If three or more drugs are used, consideration should first be given to organizing them into two drugs.

Here, patients should be made aware of the two options: converting two antipsychotics to monotherapy or continuing with two antipsychotics. Whichever option is chosen, the importance of continuation of the antipsychotic should be fully explained. It is also important to initiate and continue the 'What you can do in your daily life to achieve recovery' approach, as indicated in Appendix 2, in order to maintain remission (recovery).

【Step 2】Reviewing pros and cons of each treatment option (P22)

Patients will be asked to review the pros and cons of each option. Appendix 4 and Appendix 5 are also referred to as necessary to gain an understanding of the characteristics of the side effects of antipsychotic drugs.

【Step 3】Comparing the consequences of each treatment option (P23)

A pictogram is used to help patients understand the effects of each option (what happens as a result of choosing each option).

It is based on the results of a meta-analysis[2] of five randomized controlled trials comparing dose reduction and dose maintenance from high-dose antipsychotics (>600 mg chlorpromazine equivalent/day). The primary outcome was treatment discontinuation due to all causes, and the pictogram shows the proportion of patients per option who did not discontinue across the five trials (study duration: 1 month to 1 year), the estimated proportion of patients who would have continued treatment was 121/151 (80.1%) in the dose reduction group and 105/117 (89.7%) in dose maintenance group, with no statistically significant difference in treatment discontinuation rates between the two groups (risk ratio: 1.59, 95% confidence interval: 0.85-2.94, $p = 0.14$).

However, only a few studies have examined the effect of antipsychotic dose reduction, and further studies may change the statistical significance. Currently, evidence is only available for relatively short-term outcomes of ≤1 year.

【Step 4】What is important to you? (Value clarification exercise) (P24)

In this section, we discuss with the patient to clarify his or her values and preferences regarding each option. First, the patient is asked to rate the importance of the pros and cons of each of the options discussed in Step 2. Then, during the consultation, the patient is specifically asked to explain why he or she marked the number 0–5. In other words, this is a step to organize and share subjective information such as the patient's values and preferences together for shared decision-making, rather than simply automatically selecting the option with the highest score. Other items freely described by the patient should also be asked for details and used as material for shared decision-making.

【Step 5】Preparation for a consultation discussion (P25)

Ask the patient to proactively answer questions and concerns regarding the two options. Based on the information provided in steps 3 and 4, we discuss whether to continue polypharmacy or shift to monotherapy.

References

1. Gardner DM, Murphy AL, O'Donnell H, Centorrino F, Baldessarini RJ. International consensus study of antipsychotic dosing. *Am J Psychiatry.* 2010 Jun;167(6):686-93. doi: 10.1176/appi.ajp.2009.09060802.
2. Tani H, Takasu S, Uchida H, Suzuki T, Mimura M, Takeuchi H. Factors associated with successful antipsychotic dose reduction in schizophrenia: a systematic review of prospective clinical trials and meta-analysis of randomized controlled trials. *Neuropsychopharmacology.* 2020 Apr;45(5):887-901. doi: 10.1038/s41386-019-0573-7.
3. Matsui K, Tokumasu T, Takekita Y, Inada K, Kanazawa T, Kishimoto T, Takasu S, Tani H, Tarutani S, Hashimoto N, Yamada H, Yamanouchi Y, Takeuchi H. Switching to antipsychotic monotherapy vs. staying on antipsychotic polypharmacy in schizophrenia: A systematic review and meta-analysis. *Schizophr Res.* 2019 Jul;209:50-57. doi: 10.1016/j.schres.2019.05.030.
