# Supplementary material for: Development and acceptability testing of a decision aid for considering whether to reduce antipsychotics in individuals with stable schizophrenia
Source: Neuropsychopharmacol Rep. 2023 Jul 14;43(3):391–402. doi: 10.1002/npr2.12366 (PMC10496039; doi:10.1002/npr2.12366)
Supplement: Supplementary file 5 — Appendix S5 [file NPR2-43-391-s006.pdf]

継続

減薬

今後の治療法を一緒に決めるための手引き

抗精神病薬

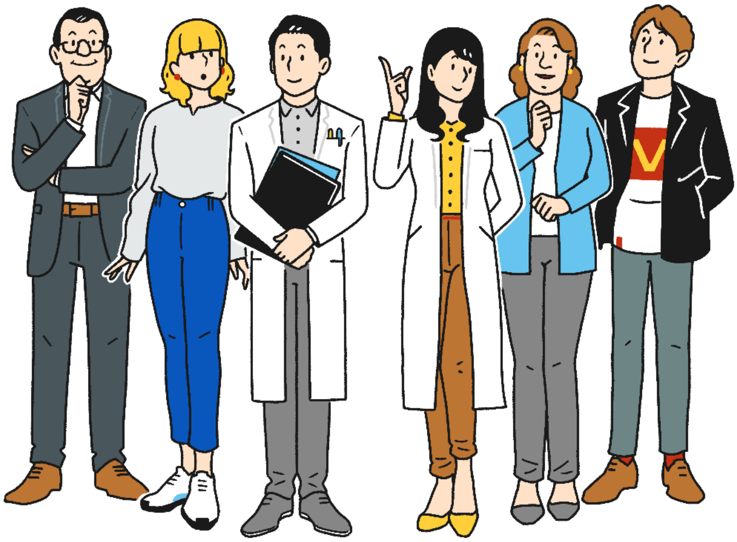

この手引きは**「向精神薬の出口戦略ガイドライン」**にもとづいて作成されています

この手引きについて 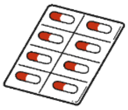

　この手引きは、統合失調症で抗精神病薬を使用中の方が、この先の治療について医療者と一緒に決めるためのものです。

　抗精神病薬はずっと継続服用することが推奨されます。減量することが良いことも良くないこともあります。そこで、抗精神病薬を減薬するかしないか、減薬する場合には、薬の量を減らすか、薬の種類を減らすか、それぞれの長所・短所を比較しながら、自分に合った方法を医療者と一緒に選ぶために、この手引きを作成しました。

【この手引きの対象となる方】

・統合失調症の方で、抗精神病薬を服用中の方

【この手引きの対象とならない方】

・抗精神病薬以外の薬の減薬方法をお探しの方
・症状が安定していない方
・抗精神病薬を3種類以上服用中の方

ご自身がこの手引きの対象となるかわからない方は
医師に相談しましょう

もくじ

この手引きの使い方 3

統合失調症とは 4

この手引きで推奨しないこと・扱わないこと 5

この先の治療の選択肢 8

A

選択肢1 『1つの抗精神病薬の量を減らす』 10

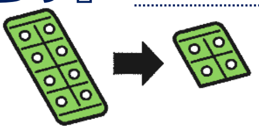

選択肢2 『1つの抗精神病薬の量を減らさず そのままの量で服用する』 12

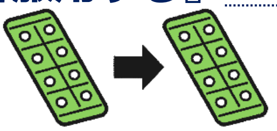

B

選択肢1『抗精神病薬の種類を1つに減らす』 19

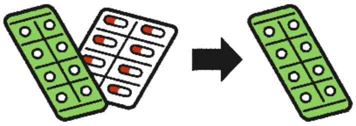

選択肢2『抗精神病薬の種類を減らさず 2つのまま服用する』 20

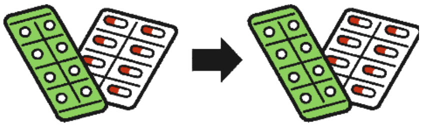

巻末付録

付録1 統合失調症治療で目指すこと（リカバリー）とは？ 27

付録2 日常生活でリカバリーに向けてできること 具体例 28

付録3 クロルプロマジン換算600mg/日 換算表 29

付録4 副作用について知る 31

付録5 抗精神病薬の種類と副作用に関係した特徴 32

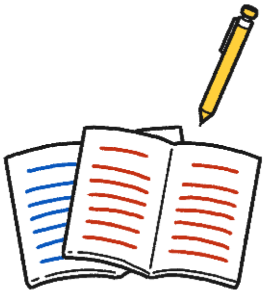

この手引きの使い方

　この手引きは、医療者と話し合いながら、この先の治療法を選ぶためのものです。この手引きを自宅に持ち帰ってよく読み、ご家族などに相談するなどし、十分に検討しながら、今後の治療の方針を一緒に考えていきます。

手引きをよく読みます
〇をつけたりメモ欄に
記入したりします

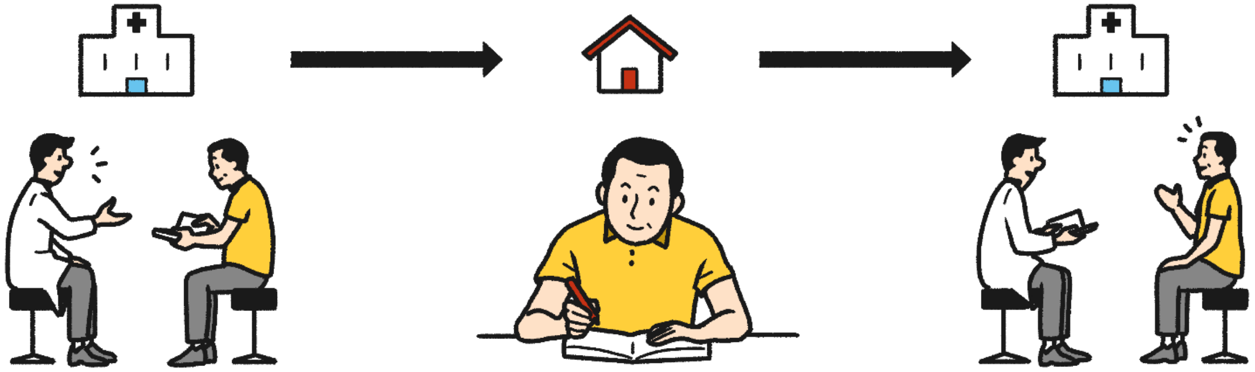

診察で

・現在の状態
・治療の選択肢
　について確認します

診察で

・〇 をつけた項目や
　メモした内容について
　話し合います
・今後の方針を決めます

※一度で決まらない場合は
また持ち帰って検討します

統合失調症とは

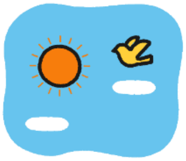

　統合失調症は、思春期から青年期に始まることが多く、幻覚や妄想などの症状が出現し、社会生活や日常生活に大きな影響がでる病気です。

　希望を持って本人の望む生活を送ること（リカバリー）が治療の目標となります。リカバリーの実現のためには、薬物療法と心理社会的治療の両方ともが必須であると考えられています。

　薬物療法の中心は抗精神病薬というグループのお薬で、幻覚や妄想を抑えることに高い効果があることが知られています。理想的には抗精神病薬を、単剤で、適切な用量で、服用することが推奨されています。単剤とは、抗精神病薬を１種類だけ使用することです。適切な用量は人によって異なり、この手引きではクロルプロマジン換算という計算方法で、1日600mg以下という量を目安にしています。

　この手引きは２種類の抗精神病薬を１種類に減らしたい、またはクロルプロマジン換算で1日600mg以上のお薬をそれ以下に減らしたい、という方のためのものです。

この手引きで扱わないこと・推奨しないこと

- **統合失調症の診断が定まっていない方については扱いません**

　統合失調症は、治療が行われない場合に幻覚や妄想が6ヶ月以上持続する病気のことをいいます。

　統合失調症は一般的に前駆期(症状が出始めの時期)、急性期(症状が活発な時期)、維持期(抗精神病薬により症状が落ち着く時期)に分けることが多いです。この手引きは **維持期の患者さんを扱っています**。

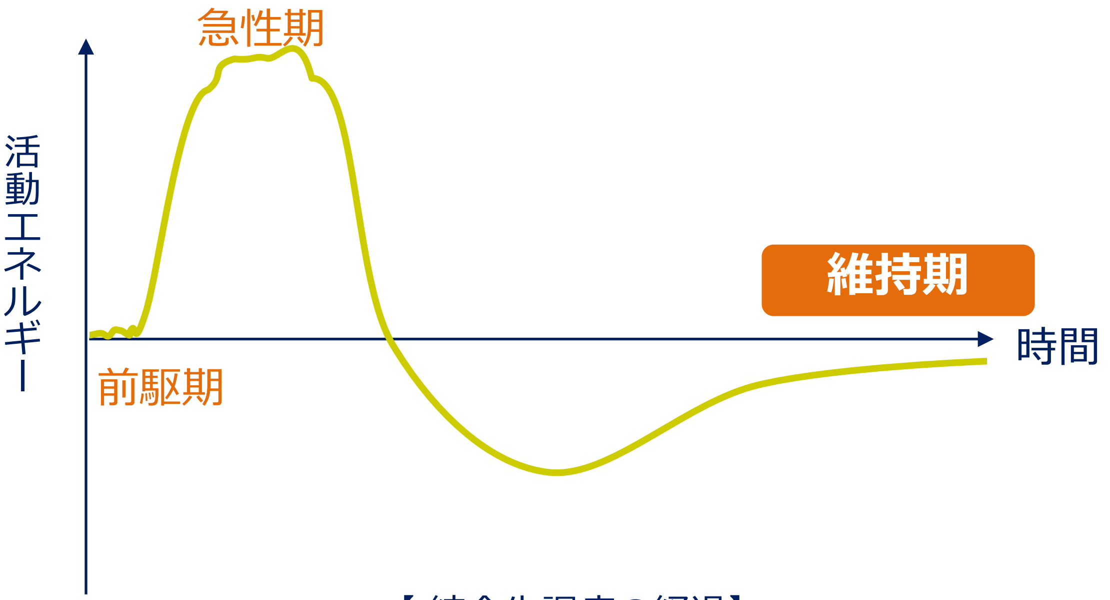

【統合失調症の経過】

この手引きで扱わないこと・推奨しないこと
つづき

- **症状が落ち着いていない方の減薬は推奨しません**

　抗精神病薬の量や種類を減らすと、一定の割合で統合失調症の症状が悪化することが知られています。したがって、現在の病気の症状が落ち着いていない方が抗精神病薬の量や種類を減らすことは、この手引きでは推奨しません。

- **３種類以上の抗精神病薬を服用中の方については扱いません**

　３種類以上の抗精神病薬を一度に飲むことは一般的ではなく、研究もほとんどないため、この手引きでは扱いません。３種類以上の抗精神病薬を内服中で、量や種類を減らしたい方は、なぜ３種類以上の抗精神病薬が必要なのかについて、主治医と相談してみて下さい。

- **抗精神病薬を飲まないこと（休薬・断薬）は推奨しません**

　抗精神病薬を飲まないこと（休薬・断薬）は、病状を悪化させ、リカバリーを困難にする場合が多いことが知られています。したがって、この手引きでは推奨しません。

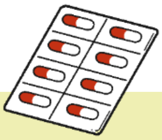

この手引きで扱わないこと・推奨しないこと
つづき

● 抗精神病薬以外の薬については扱いません

抗精神病薬以外に精神科で使用されるお薬には、睡眠薬、抗不安薬、抗うつ薬、抗けいれん薬、気分安定薬（躁うつ病の気分の波を改善する）などのお薬のグループがあります。これらは、統合失調症の本来の症状を改善するためのお薬ではなく、これらのお薬の併用は、この手引きでは扱いません。抗精神病薬以外の精神科のお薬を内服中で、量や種類を減らしたい方は、なぜこれらの薬が必要なのかについて、主治医と相談してみて下さい。

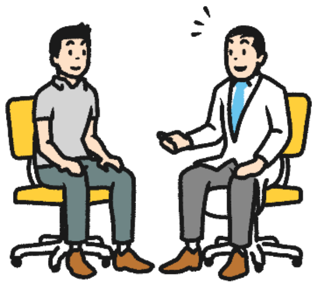

つぎは、この手引きの対象となる方のこの先の
治療の選択肢をみていきます ☞

この先の治療の選択肢

この先の治療の選択には **A** ・ **B** 2つのパターンがあり、それぞれに2つの選択肢があります

A

選択肢 1

『1つの抗精神病薬の量を減らす』

- 例えば、8錠から4錠に減らす

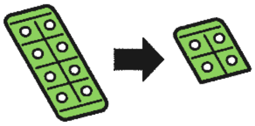

選択肢 2

『1つの抗精神病薬の量を減らさずそのままの量で服用する』

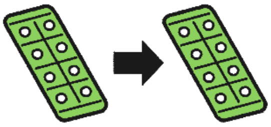

B

選択肢 1

『抗精神病薬の種類を２種類から１種類に減らす』

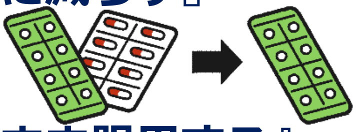

選択肢 2

『抗精神病薬の種類を減らさず２種類のまま服用する』

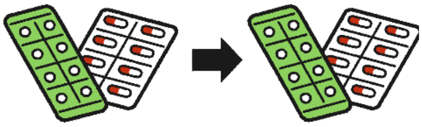

この先は **A** と **B** をわけてみていきます☞

A　この先の治療の選択肢

ステップ 1　各選択肢の内容を理解しましょう

選択肢1　『1つの抗精神病薬の量を減らす』

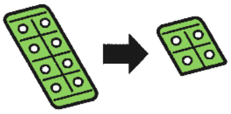

この選択肢を選んだ場合に行うこと

- 日常生活でリカバリーに向けてできることを続ける（始める）
- １つの抗精神病薬の量を減らす

選択肢2
『1つの抗精神病薬の量を減らさずそのままの量で服用する』

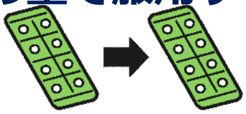

この選択肢を選んだ場合に行うこと

- 日常生活でリカバリーに向けてできることを続ける（始める）
- １つの抗精神病薬の量を減らさずそのままの量で服用する

つぎに進む前に、巻末の付録で情報の整理をしましょう

- 統合失調症治療で目指すこと（リカバリー）とは？ ➡ **付録 1**
- 日常生活でリカバリーに向けてできることの具体例 ➡ **付録 2**
- 使用している抗精神病薬の種類と量を知る ➡ **付録 3**
- 副作用について知る ➡ **付録 4**
- 抗精神病薬の種類と特徴 ➡ **付録 5**

それでは、各選択肢についてみていきます☞

A 選択肢 1

『１つの抗精神病薬の量を減らす』

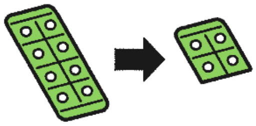

　抗精神病薬は継続して使用することが大切です。抗精神病薬を使用することで、幻聴や妄想などの精神病症状を軽減することができ、リカバリーに向けて治療を進められることが今までにわかっているからです。

　リカバリーを目指すには心身の状態が安定していることが望ましいですが、時には使用しているお薬の量が多くなってしまう、あるいは使用するお薬の種類が多くなってしまう（ B パターン）方もいらっしゃいます。

　病状や経過によって様々ですが、症状は安定しているのだが、副作用が強く出てしまっている場合などでは、お薬の量を減らす事によって副作用が減る可能性があります。

A 選択肢 1

『１つの抗精神病薬の量を減らす』つづき

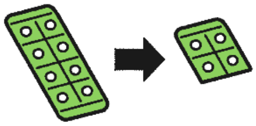

現在のところ、どのようにお薬を減らせば良いということは残念ながら明らかになっていませんので、後に述べるような長所と短所（14ページ）や巻末の **付録** などを参考にしていただき、お薬を減らす方法やペースについては主治医と相談のうえ始めてください。

※現在どのようなお薬をどれ位の量で使用しているかを知っておくことは、とても大切です。気になる方は、巻末 **付録3** を参考のうえ主治医に確認してみてください。

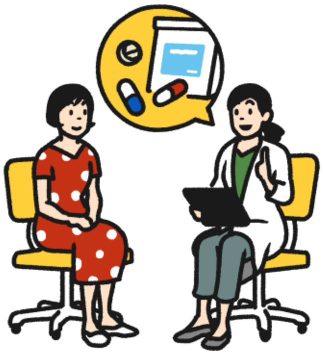

A 選択肢 2

『１つの抗精神病薬の量を減らさず　そのままの量で服用する』

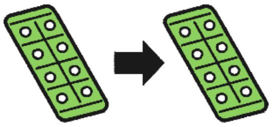

この選択肢に興味を持たれた方は、現在のところ心身の状態も安定し、リカバリーに向けて少しずつ前進されている、あるいは落ち着いて毎日を過ごされている方などなのかもしれません。

<u>抗精神病薬は継続して使用することが大切です</u>。

　また、お薬の効果や副作用を正しく理解するために、適切な量の抗精神病薬で治療をしていくことが大切といわれています。

　一方、これまでの治療の経過から、お薬の量が多くなっている場合もあるかもしれません。しかしながら、心身の状態を安定させてくれる可能性もある反面で、副作用が出やすくなってしまう心配があります。

A 選択肢 2

『１つの抗精神病薬の量を減らさず そのままの量で服用する』 つづき

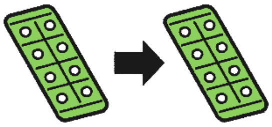

そのままの量で治療を続けることを望まれている方も、後に述べるような長所と短所（14ページ）や巻末の **付録** などを参考に、お薬の飲み方や剤形（錠剤、散剤、液剤や注射剤などのお薬のタイプ）などについて、主治医に相談してみても良いかもしれません。

※現在どのようなお薬をどれ位の量で使用しているかを知っておくことはとても大切です。気になる方は、巻末の **付録3** を参考のうえ主治医に確認してみてください。

つぎは、各選択肢の長所・短所を比較してみましょう ☞

A 各選択肢の長所・短所

ステップ 2 各選択肢の長所・短所を比較してみます

	選択肢1<br>『1つの抗精神病薬の量を減らす』	選択肢2<br>『1つの抗精神病薬の量を減らさず そのままの量で服用する』
長所 ☺	● 副作用が減るかもしれない（錐体外路症状，自律神経症状）<br>● 薬の量が多くなるにつれて増えると言われている副作用が減るかもしれない（認知機能障害，心臓突然死）<br>● 副作用が減って体を動かしやすくなるかもしれない<br>● 薬の費用が減るかもしれない	● 症状が変わらない<br>● 今の安定した状態が維持できる
短所 ☹	● 症状が悪化するかもしれない<br>● 症状が悪化することで入院が必要になるかもしれない	● 副作用の心配があるかもしれない（錐体外路症状，自律神経症状）<br>● 薬の量が多くなるにつれて増えると言われている副作用の心配があるかもしれない（認知機能障害，心臓突然死）<br>● 薬の費用がかかるかもしれない

A 各選択肢を選んだ結果

ステップ3 各選択肢を選んだ結果を比較してみます

統合失調症の薬物療法では、継続できることがとても大切です。選んだ結果が治療継続にどのように影響を与えるのか、研究結果[1-5]を示しました。抗精神病薬を使用中の人が各選択肢を選んだ場合、どの位の割合で**「問題なく治療を継続できるか」**を示しました。

	**選択肢1**<br>**『1つの抗精神病薬の量を減らす』**	**選択肢2**<br>**『1つの抗精神病薬の量を減らさず そのままの量で服用する』**
1ヶ月〜1年後	5つの試験（1ヶ月〜1年）で問題なく治療を継続できた割合は、**151人中121人（80.1%)**でした<br>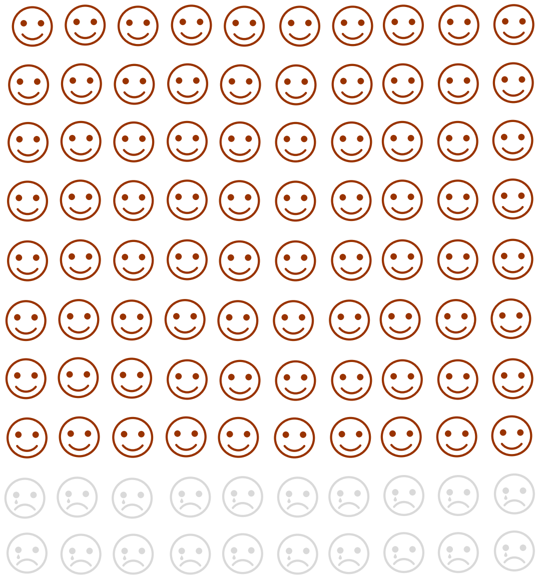	5つの試験（1ヶ月〜1年）で問題なく治療を継続できた割合は、**117人中105人（89.7%)**でした<br>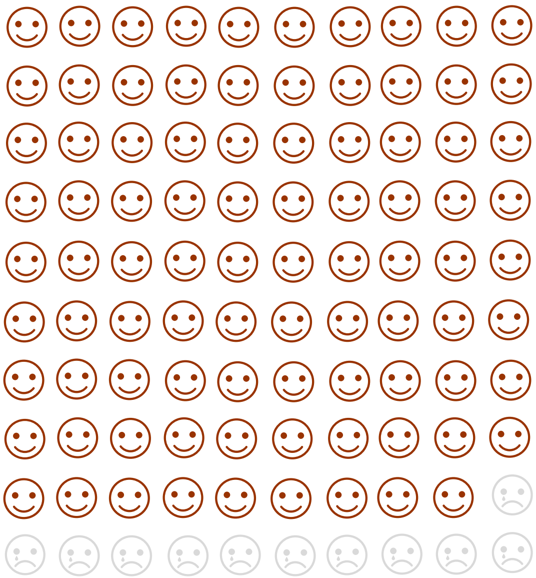
	両群間で統計学的に有意な差はありませんでした	

A 自分にとって重要なこと

ステップ 4 あなたにとって重要なことを整理します

以下に、各選択肢を選ぶおもな理由をあげました。それぞれあなたにとってどのくらい重要ですか？あてはまる数字に○をつけ、重みづけをしてみましょう。

選択肢1『1つの抗精神病薬の量を減らす』理由

	重要でない					重要である
副作用が減るかもしれない	0	1	2	3	4	5
副作用が減って体を動かしやすくなるかもしれない	0	1	2	3	4	5
その他（自由に書いてみましょう）						
・	0	1	2	3	4	5
・	0	1	2	3	4	5
・	0	1	2	3	4	5

選択肢2『1つの抗精神病薬の量を減らさずそのままの量で治療する』理由

	重要でない					重要である
症状が変わらない	0	1	2	3	4	5
今の安定した状態が維持できる	0	1	2	3	4	5
その他（自由に書いてみましょう）						
・	0	1	2	3	4	5
・	0	1	2	3	4	5
・	0	1	2	3	4	5

A 診察で話し合うための準備

ステップ 5 医師との話し合いにむけた準備をします

あなたの考えや重みづけをもとに、選択肢1『**1つの抗精神病薬の量を減らす**』選択肢2『**1つの抗精神病薬の量を減らさずそのままの量で服用する**』について、つぎの診察で話し合います。

疑問や気になったことなど、今の気持ちや考えを書いておきましょう

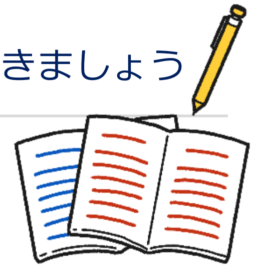

B この先の治療の選択肢

ステップ 1 各選択肢の内容を理解しましょう

選択肢1 『抗精神病薬の種類を2種類から1種類に減らす』

この選択肢を選んだ場合に行うこと

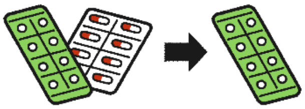

- 日常生活でリカバリーに向けてできることを続ける（始める）
- 抗精神病薬を2種類から1種類にする

選択肢2

『抗精神病薬の種類を減らさず２種類のまま服用する』

この選択肢を選んだ場合に行うこと

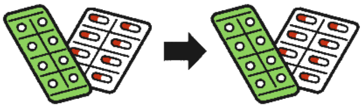

- 日常生活でリカバリーに向けてできることを続ける（始める）
- 抗精神病薬の2種類併用を続ける

つぎに進む前に、巻末の付録で情報の整理をしましょう

- 統合失調症治療で目指すこと（リカバリー）とは？ ➡ **付録1**
- 日常生活でリカバリーに向けてできることの具体例 ➡ **付録2**
- 使用している抗精神病薬の種類と量を知る ➡ **付録3**
- 副作用について知る ➡ **付録4**
- 抗精神病薬の種類と特徴 ➡ **付録5**

それでは、各選択肢についてみていきます☞

B **選択肢 1**

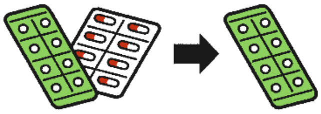

『抗精神病薬の種類を2種類から1種類に減らす』

これまでにも述べられているように、抗精神病薬は継続して使用することが大切です。

また、使用されている抗精神病薬の効果や副作用を正しく理解するためには、1種類の抗精神病薬による治療が行われることが基本となります。このため、これまでの病状や治療の経過から、2種類の抗精神病薬が使用されている場合でも、1種類の抗精神病薬にまとめていくことは大切な選択と言えるかもしれません。

ただし、このような場合でも後に述べるような長所と短所（22ページ）があります。また、使用されている抗精神病薬によっても種類を減らす方法やペースなどが異なります。巻末の **付録** などを参考に、主治医と注意すべきことを確認および相談をしてください。

＊3種類以上の抗精神病薬が使用されている場合＊

3種類以上の抗精神病薬の使用については、その治療が良いか悪いかについて、具体的に調査されたことはありません。このため、想定外のリスクが存在する可能性もあります。今後の薬物治療について、主治医を含めた医療関係者と十分な相談を行う必要があるでしょう。

B 選択肢 2

『抗精神病薬の種類を種類を減らさず 2種類のまま服用する』

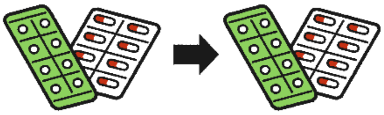

この選択肢に興味を持たれた方は、現在のところ心身の状態も安定し、リカバリーに向けて少しずつ前進されている、あるいは落ち着いて毎日を過ごされている方などなのかもしれません。

抗精神病薬は継続して使用することが大切です。

また、お薬の効果や副作用を正しく理解するためには、1種類の抗精神病薬で治療をしていくことが大切といわれています。

一方、これまでの治療の経過から、2種類の抗精神病薬が使用されている場合もあるかもしれません。しかしながら、精神症状の安定を助けている可能性もある反面で、副作用が出てきた場合に、どちらのお薬がその原因となっているか判断が難しくなる心配があります。

B 選択肢 2

『抗精神病薬の種類を種類を減らさず 2種類のまま服用する』 つづき

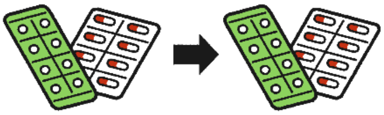

2 種類のままで治療を続けることを望まれている方も、後に述べるような長所と短所（22ページ）や巻末の **付録** などを参考に、2種類の抗精神病薬を続けるうえで注意すべきことを主治医に相談してみても良いかもしれません。

※現在どのようなお薬をどれ位の量で使用しているかを知っておくことはとても大切です。気になる方は、巻末の **付録3** を参考のうえ主治医に確認してみてください。

＊3種類以上の抗精神病薬が使用されている場合＊

3種類以上の抗精神病薬の使用については、その治療が良いか悪いかについて、具体的に調査されたことはありません。このため、想定外のリスクが存在する可能性もあります。今後の薬物治療について、主治医を含めた医療関係者と十分な相談を行う必要があるでしょう。

つぎは、各選択肢の長所・短所を比較してみましょう ☞

B 各選択肢の長所・短所

ステップ 2　各選択肢の長所・短所を比較してみます

	選択肢1<br>『抗精神病薬の種類を<br>2種類から1種類に減らす』	選択肢2<br>『抗精神病薬の種類を減らさず<br>2種類のまま服用する』
長所 ☺	● 副作用が減るかもしれない（錐体外路症状, 自律神経症状）<br>● 薬の量が多くなるにつれて増えると言われている副作用が減るかもしれない（認知機能障害, 心臓突然死）<br>● 副作用が減って体を動かしやすくなるかもしれない<br>● 薬がシンプルになり、のみやすくなる<br>● 薬の費用が減るかもしれない	● 症状が変わらない<br>● 今の安定した状態が維持できる
短所 ☹	● 1剤への移行が上手く行かないかもしれない<br>● 症状が悪化するかもしれない<br>● 症状が悪化することで入院が必要になるかもしれない	● 副作用の心配があるかもしれない（錐体外路症状，自律神経症状）<br>● 薬の量が多くなるにつれて増えると言われている副作用の心配があるかもしれない（認知機能障害, 心臓突然死）<br>● 薬の費用がかかるかもしれない<br>● 2種類の抗精神病薬の服用継続による副作用リスクの増大があるかもしれない

B 各選択肢を選んだ結果

ステップ 3 各選択肢を選んだ結果を比較してみます

統合失調症の薬物療法では、継続できることがとても大切です。選んだ結果が治療継続にどのように影響を与えるのか、研究結果[6-11]を示しました。抗精神病薬を使用中の人が各選択肢を選んだ場合、どの位の割合で**「問題なく治療を継続できるか」**を示しました。

	**選択肢1**<br>**『抗精神病薬の種類を 2種類から1種類に減らす』**	**選択肢2**<br>**『抗精神病薬の種類を減らさず 2種類のまま服用する』**
1ヶ月〜1年後	6つの試験（1ヶ月〜1年）で、悪いことがなく治療を継続できた割合は**177人中120人 (67.8%)**でした<br>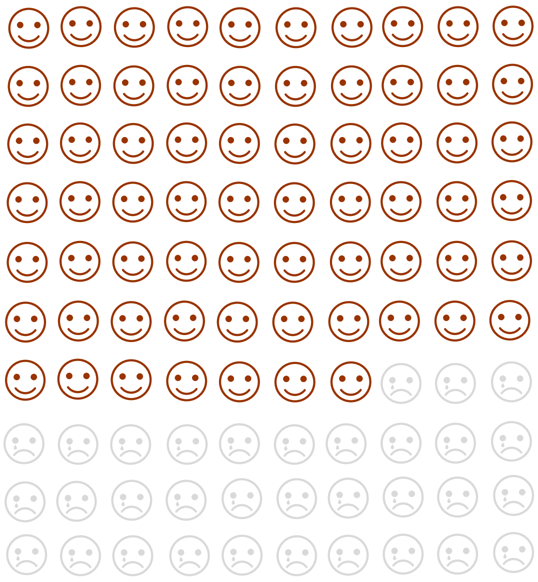	6つの試験（1ヶ月〜1年）で、悪いことがなく治療を継続できた割合は **164人中140人 (85.4%)** でした<br>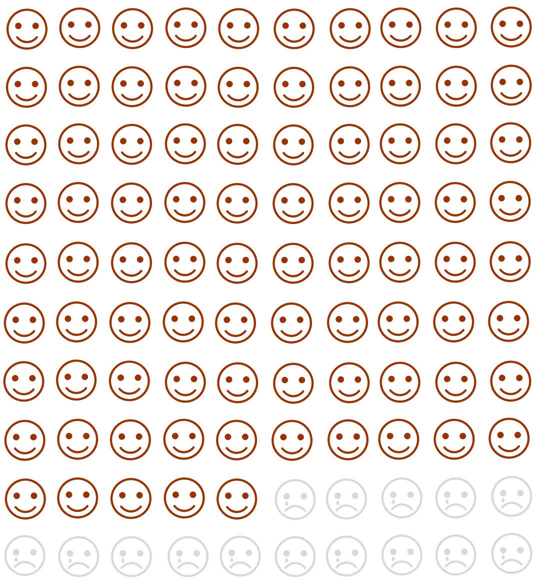

両群間に統計学的に有意な差がありました

B 自分にとって重要なこと

ステップ 4　あなたにとって重要なことを整理します

以下に、各選択肢を選ぶおもな理由をあげました。それぞれあなたにとってどのくらい重要ですか？あてはまる数字に○をつけ、重みづけをしてみましょう。

選択肢1『抗精神病薬の種類を2種類から1種類に減らす』理由

	重要でない					重要である
副作用が減るかもしれない	0	1	2	3	4	5
副作用が減って体を動かしやすくなるかもしれない	0	1	2	3	4	5
その他（自由に書いてみましょう）						
・	0	1	2	3	4	5
・	0	1	2	3	4	5
・	0	1	2	3	4	5

選択肢2『抗精神病薬の種類を減らさず2種類のまま服用する』理由

	重要でない					重要である
症状が変わらない	0	1	2	3	4	5
今の安定した状態が維持できる	0	1	2	3	4	5
その他（自由に書いてみましょう）						
・	0	1	2	3	4	5
・	0	1	2	3	4	5
・	0	1	2	3	4	5

B 診察で話し合うための準備

ステップ 5　医師との話し合いにむけた準備をします

あなたの考えや重みづけをもとに、**選択肢1『抗精神病薬の種類を2種類から1種類に減らす』選択肢2『抗精神病薬の種類を減らさず2種類のまま服用する』**について、つぎの診察で話し合います。

疑問や気になったことなど、今の気持ちや考えを書いておきましょう

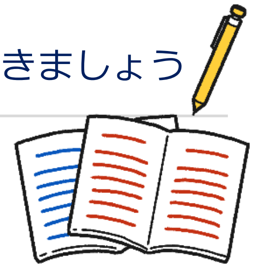

付録1 統合失調症治療で目指すこと（リカバリー）とは？

付録2 日常生活でリカバリーに向けてできること 具体例

付録3 クロルプロマジン換算600mg/日 換算表

付録4 副作用について知る

付録5 抗精神病薬の種類と副作用に関係した特徴

付録1　統合失調症治療で目指すこと（リカバリー）とは？

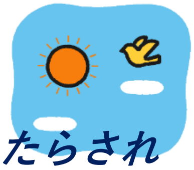

『リカバリー(Recovery)』とは，疾患によりもたらされた制限が生活の中にあったとしても，満足感のある，希望に満ちた，人の役に立つ人生を生きること，精神疾患のもたらすつらく悲しい影響を乗り越えて成長し，人生に新しい意味や目的を見出すことを意味します． *（W. A. Anthony）*

　統合失調症治療で目指すものとして『リカバリー』という一つの考え方があることはご存知でしょうか。決して望んだ訳ではない病を抱えて生活を続ける中で、『どうなりたいか』を考えることや目指すことはとても大切なことですが、リカバリーという考え方がヒントになるかもしれません。

　リカバリーには様々な考え方がありますが、次ページの図のように『臨床的リカバリー』と『パーソナルリカバリー』の二つの側面があると言われています。つまり、病気の状態を安定させ、症状や機能を改善し維持することとその人自身の希望する人生に向けて近づいていくことの双方が大切なのだという考え方です。

　臨床的リカバリーとは、Liberman博士らの定義によると①症状の寛解、②就労ないし就学（半日程度以上）、③自立した生活（金銭面、治療面ともに）④社会的人間関係の構築（週1回以上の友人との付き合い）のすべてを2年以上持続すること、とあります。

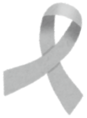

付録1 統合失調症治療で目指すこと（リカバリー）とは？ つづき

しかしながら、そのことだけが重要なわけではなく、まさに上に示したとおりにその人自身が人生に意味や目的を見出すプロセスが『パーソナルリカバリー』であり、専門家をはじめ大切な人達と相談し、議論し、時には悩みながらも、ともにリカバリーを目指していけると良いのではないでしょうか。

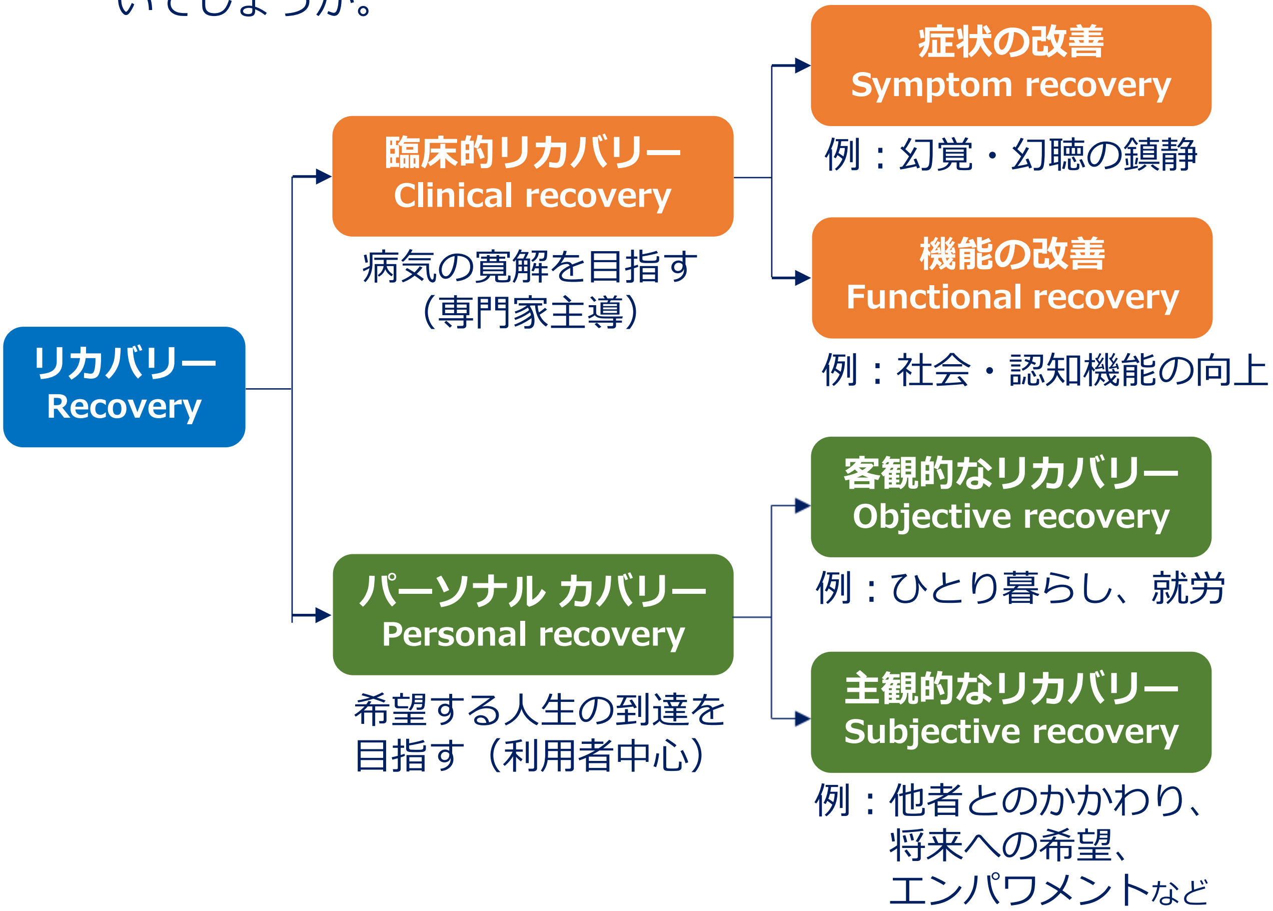

山口創生：医学のあゆみ 261(10), 941-8, 2017 より引用[15)]

※ パーソナルリカバリーの構成要素（Leamyら，2011）[16)]
①他者との関わり、②将来への希望、③アイデンティティの確立、
④生活の意義、⑤エンパワメント

付録2 日常生活でリカバリーに向けてできること具体例

ここでは日常生活でリカバリーに向けてできることの具体例をあげてみました。ぜひ参考にしてみてください。

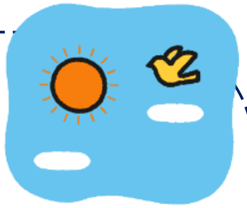

- **自分の望む生活や希望をもつ**
- **楽しいと思える時間をもつ**
- **自分の生活について決めている感覚を持てる**
- **希望を言葉にすることができる**
- **誰かの役にやっている感覚を持てる**
- **規則正しい生活をする**
- **十分な睡眠をとる**
- **日中に自分の居場所がある**
- **相談できる相手（友人、家族、支援者など）や仲間がいる**
- **生活に必要な支援を知る・利用する**
- **治療に必要な支援を知る・利用する**
- **就労などの社会参加に必要な支援を知る・利用する**
- **病気について知る**
- **自分の状態や症状を知る**
- **悪化のサイン、対処の方法について知る**
- **非常時に「誰に」連絡するかを決める**

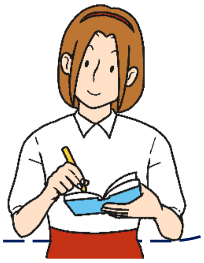

付録3 クロルプロマジン換算600mg/日 換算表

これはクロルプロマジン600mg/日と等価換算（おおよそ同じ強さ）とされるお薬の量の一覧表です。いま飲んでいるお薬がここに書かれている量よりも多い方がこの手引きの対象になります。

1. 飲み薬の方

一般名	代表的な商品名	等価換算量
アセナピン	シクレスト	15mg*
アリピプラゾール	エビリファイ	30mg
オランザピン	ジプレキサ	20mg
クエチアピン	セロクエル	750mg
クロザピン	クロザピン	400mg
**クロルプロマジン**	**コントミン**	**600mg**
スルトプリド	バルネチール	1200mg*
スルピリド	ドグマチール	800mg
ゾテピン	ロドピン	300mg
チアプリド	グラマリール	600mg*
チミペロン	トロペロン	7.8mg*
ネモナプリド	エミレース	27mg*
パリペリドン	インヴェガ	9mg
ハロペリドール	セレネース	10mg
ピモジド	オーラップ	8mg
フルフェナジン	フルメジン	12mg
ブレクスピプラゾール	レキサルティ	記載なし
ブロナンセリン	ロナセン	24mg*
プロペリシアジン	ニューレプチル	120mg*
ブロムペリドール	インプロメン	12mg*
ペルフェナジン	ピーゼットシー	30mg
ペロスピロン	ルーラン	48mg*
リスペリドン	リスパダール	6mg
レボメプロマジン	レボトミン	400mg

Gardnerらの等価換算表[12]を一部改変

付録3 クロルプロマジン換算600mg/日 換算表

つづき

2. 注射の方

一般名	代表的な商品名	等価換算量
アリピプラゾール 持効性注射剤	エビリファイ 持効性注射剤	600mg (4週ごと) *
パリペリドン パルミチン酸エステル	ゼプリオン	112.5mg (4週ごと) *
ハロペリドール デカン酸エステル	ハロマンス	150mg (4週ごと)
フルフェナジン デカン酸エステル	フルデカシン	50mg (4週ごと)
リスペリドン 持効性注射剤	リスパダール コンスタ	50mg (2週ごと)

Gardnerらの等価換算表[12]を一部改変

注：この表はGardnerらの論文(2010年)の等価換算表[12)]に基づいて作成されています。ただし、*の印があるお薬はGardnerの論文に記載がなかったので、稲垣・稲田の換算表[13,14)]に基づいた値をのせています。

付録4 副作用について知る

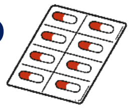

統合失調症の薬物治療では、抗精神病薬を続けて使用することは、とても大切です。しかしながら、お薬には副作用として下に示すような症状が出てくる場合があります。

もし、下に示すような副作用が見られる場合は主治医にご相談ください。

食欲が増えすぎて困っている/ 体重が増えてしまう
口が渇いて仕方がない / 水を沢山飲んでしまう
便秘 / 尿が出にくい
目の焦点が合わない / 視界がぼやけてしまう
生理が来ない / 乳汁が出てしまう
勃起しない / 性欲が湧かない
じっと座っていることができない
足がムズムズして、ついウロウロとしてしまう
口や手が勝手に動いてしまう / 手や指が震える
舌がもつれて、うまくお話できない
ヨダレや唾の量が多くなっている
体が強張ってしまう、体がうまく動かせない
眠気が強い、常にぼんやりする / 立ちくらみがする

他にも、気になる症状がある方は遠慮せずに主治医にご相談ください。

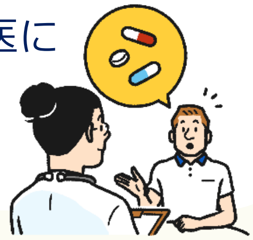

付録5 抗精神病薬の種類と副作用に関係した特徴

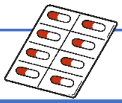

副作用	頻度	抗精神病薬名
急な薬剤中止が難しい便秘や口の渇きなどが出る	高	クロルプロマジン、クロザピン[1]
	中	ハロペリドール、オランザピン、パリペリドンクエチアピン、リスペリドン
眠気が増える	高	クロルプロマジン、クロザピン[1]<br>オランザピン、クエチアピン
	中	ハロペリドール、ルラシドン<br>パリペリドン、リスペリドン
体重が増える	高	クロルプロマジン、クロザピン[1]<br>オランザピン、クエチアピン<br>パリペリドン、リスペリドン
	中	アセナピン、ブレクスピプラゾール<br>ハロペリドール、スルピリド
アカシジア[2]が出る	高	ハロペリドール
	中	アリピプラゾール、アセナピン<br>ブレクスピプラゾール、クロルプロマジン<br>ルラシドン、リスペリドン、スルピリド
パーキンソン症状[3]が出る	高	クロルプロマジン、ハロペリドール
	中	ルラシドン、パリペリドン、リスペリドン<br>スルピリド
血圧が下がる	高	クロルプロマジン、クロザピン[1]、パリペリドンクエチアピン、リスペリドン
	中	ハロペリドール、オランザピン
プロラクチン[4]が増える	高	クロルプロマジン、ハロペリドール<br>パリペリドン、リスペリドン、スルピリド
	中	アセナピン、オランザピン

モーズレー処方ガイドライン 13版[17] 一部改変

代表的な商品名については付録1等を参照ください

1.治療抵抗性（抗精神病薬を2種類以上十分量、十分期間使用しても症状の改善が見られないなどのこと）の患者さんにのみ使用する

2. おもに足のむずむず感やそわそわ感を生じること

3.パーキンソン症状とは「動作が遅くなった」「声が小さくなった」「表情が少なくなった」「歩き方が ふらふらする」「歩幅がせまくなった（小刻み歩行）」「一歩目が出ない」「手が震える」「止まれず走り出すことがある」「手足が固い」などの症状のこと

4.プロラクチンというホルモンが増えることで女性では月経異常、男性は性欲減退や乳房の肥大が起きる場合がある

　表にあげた以外にも日本で統合失調症に使用することの出来る抗精神病薬があります。そのお薬がどのような特徴を持つかは主治医に尋ねてください。

おわりに

●自分にあった治療法を選ぶために

　治療の選択肢には、それぞれ長所と短所があります。この手引きは、それらをよく理解し、自分にとって重要なことを明らかにしながら医療者と話し合い、あなたに合った選択ができるよう作られています。

●手引きの開発プロセス

　この手引きに掲載した情報は、統合失調症の治療を経験されたことのある方々の声や意見を反映させました。精神科の専門家のチェックも受けています。また、この手引きは厚生労働科学研究費補助金・障害者政策総合研究事業「平成29年度～30年度：向精神薬の処方実態の解明と適正処方を実践するための薬物療法ガイドラインに関する研究（H29-精神-一般-001）」および「平成31年/令和1年度～2年度：向精神薬の適切な継続･減量･中止等の精神科薬物療法の出口戦略の実践に資する研究（19GC1012）」により作成しました。特定の企業からの援助は受けていません。

●手引きの更新

　この手引きは、必要に応じて見直しと更新をおこないます。

※ ここに掲載された情報は、医療者と話し合いながら対処法や治療法を決める際の手引きとなるものであり、医療者のアドバイスの代わりになるものではありません。

文献

1) Yamanouchi Y, Sukegawa T, Inagaki A, Inada T, Yoshio T, Yoshimura R, Iwata N. Evaluation of the individual safe correction of antipsychotic agent polypharmacy in Japanese patients with chronic schizophrenia: validation of safe corrections for antipsychotic polypharmacy and the high-dose method. Int J Neuropsychopharmacol. 2014;18(5).
2) Uchida H, Suzuki T, Yamazawa R, Tomita M, Nemoto T, Kimura Y, Watanabe K, Imasaka Y, Kashima H. Reducing the dose of antipsychotic agents ameliorates visual hypersensitivity attack: an ideal treatment option in terms of the adverse effect. J Clin Psychopharmacol. 2006; 26(1):50-5.
3) Volavka J, Cooper TB, Czobor P, Lindenmayer JP, Citrome LL, Mohr P, Bark N. High-dose treatment with haloperidol: the effect of dose reduction. J Clin Psychopharmacol. 2000; 20(2):252-6.
4) Hirschowitz J, Hitzemann R, Piscani K, Burr G, Frecska E, Culliton D, Mann M, Curtis C. The Dose Reduction in Schizophrenia (DORIS) Study: a final report. Schizophr Res. 1997;23(1):31-43.
5) Newcomer JW, Riney SJ, Vinogradov S, Csernansky JG. Plasma prolactin and homovanillic acid as markers for psychopathology and abnormal movements after neuroleptic dose decrease. Psychopharmacol Bull. 1992;28(1):101-7.
6) Essock SM, Schooler NR, Stroup TS, McEvoy JP, Rojas I, Jackson C, Covell NH. Effectiveness of Switching From Antipsychotic Polypharmacy to Monotherapy. Am J Psychiatry. 2011;168(7):702–8.
7) Constantine RJ, Andel R, McPherson M, Tandon R. The Risks and Benefits of Switching Patients with Schizophrenia or Schizoaffective Disorder from Two to One Antipsychotic Medication: A Randomized Controlled Trial. Schizophrenia Research. 2015;166(1–3):194–200.
8) Hori H, Yoshimura R, Katsuki A, Sugita AI, Atake K, Nakamura J. Switching to Antipsychotic Monotherapy Can Improve Attention and Processing Speed, and Social Activity in Chronic Schizophrenia Patients. J Psychiatr Res. 2013;47(12):1843–48.
9) Repo-Tiihonen E, Hallikainen T, Kivistö P, Tiihonen J. Antipsychotic Polypharmacy in Clozapine Resistant Schizophrenia: A Randomized Controlled Trial of Tapering Antipsychotic Co-Treatment. Mental Illness. 2012; 4(1):1–4.
10) Borlido C, Remington G, Graff-Guerrero A, Arenovich T, Hazra M, Wong A, Daskalakis ZJ, Mamo DC. Switching from 2 Antipsychotics to 1 Antipsychotic in Schizophrenia: A Randomized, Double-Blind, Placebo-Controlled Study. J Clin Psychiatry. 2016;77(1):e14-20.
11) Yoon HW, Lee JS, Park SJ, Lee SK, Choi WJ, Kim TY, Hong CH, Seok JH, Park IH, Son SJ, Roh D, Kim BR, Lee BO. Comparing the Effectiveness and Safety of the Addition of and Switching to Aripiprazole for Resolving Antipsychotic-Induced Hyperprolactinemia: A Multicenter, Open-Label, Prospective Study. Clin Neuropharmacol. 2016;39(6):288–94.
12) Gardner DM, Murphy AL, O'Donnell H, Centorrino F, Baldessarini RJ. International consensus study of antipsychotic dosing. Am J Psychiatry. 2010;167:686-93.
13) 稲垣中，稲田俊也：臨床精神薬理 20： 89-97, 2017
14) 稲垣中，稲田俊也：臨床精神薬理 18：1475-1480, 2015
15) 山口創生, 熊倉陽介：医学のあゆみ 261：941-948, 2017
16) Leamy M, Bird V, Le Boutillier C, Williams J, Slade M. Conceptual framework for personal recovery in mental health: systematic review and narrative synthesis. Br J Psychiatry. 2011;199:445-52.
17) Taylor DM, et al. 著. 内田裕之等訳. モーズレイ処方ガイドライン 第13版.ワイリー・パブリッシング・ジャパン.2019.

作成・向精神薬出口戦略マニュアル研究班・抗精神病薬グループ

作成：2023年3月　　更新予定日：2025年3月

この手引きは「向精神薬の出口戦略ガイドライン」にもとづいて作成されています

©2023,向精神薬出口戦略マニュアル研究班

無断複写・無断転載はご遠慮ください
