# Supplementary material for: Development and acceptability testing of a decision aid for considering whether to reduce antipsychotics in individuals with stable schizophrenia
Source: Neuropsychopharmacol Rep. 2023 Jul 14;43(3):391–402. doi: 10.1002/npr2.12366 (PMC10496039; doi:10.1002/npr2.12366)
Supplement: Supplementary file 6 — Appendix S6 [file NPR2-43-391-s003.pdf]

CONTINUING REDUCING

A **decision aid** for considering whether to reduce antipsychotics

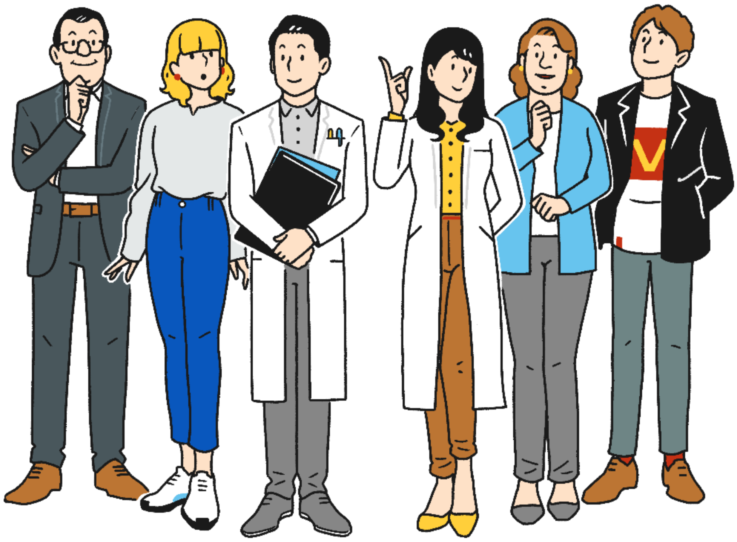

This decision aid is based on **the Guidelines for Psychotropic Drug Discontinuation Strategies**.

About this decision aid 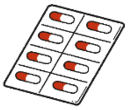

This decision aid is designed to assist individuals with schizophrenia who are using antipsychotics, to determine their further treatment with healthcare providers. Antipsychotics are recommended for continuous use for preventing relapse. Reducing antipsychotics has both pros and cons. Therefore, we have prepared this aid to help the individuals and their healthcare providers choose the appropriate method, while comparing the pros and cons of reducing or not reducing antipsychotics, and, when reducing, whether to reduce the dose or type of medication.

【Those who are eligible for this aid】

・Individuals with schizophrenia who are taking antipsychotics.

【Those who are NOT eligible for this aid】

・Individuals who are looking for a method to reduce their medication other than antipsychotics.

・Individuals with schizophrenia whose symptoms are not stable.

・Individuals who are taking more than three antipsychotics.

If you are not sure if you are eligible for this aid, please consult your doctor.

Table of Contents

How to use this decision aid? 3

What is schizophrenia? 4

What this aid does NOT recommend or address 5

Further treatment options 8

A For antipsychotic monotherapy,

Option1 **Reducing the dose of one antipsychotic** 10

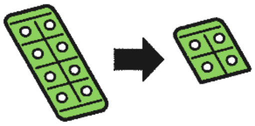

Option2 **Taking one antipsychotic at the same dose without reducing the dose** 12

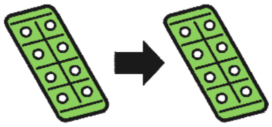

B For the combined use of two antipsychotic drugs,

Option1 **Shifting to monotherapy** 19

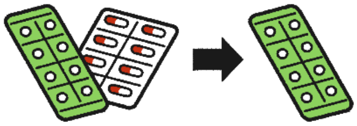

Option2 **Continuing polypharmacy** 20

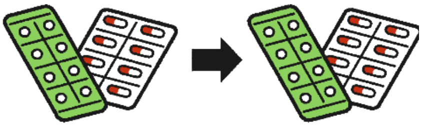

Appendices

Appendix1
What is the goal (recovery) in schizophrenia treatment? 27

Appendix2
What you can do in your daily life to achieve recovery 28

Appendix3
Chlorpromazine equivalent dose conversion table 29

Appendix4
Knowing about side effects 31

Appendix5
Types of antipsychotics and related side effects 32

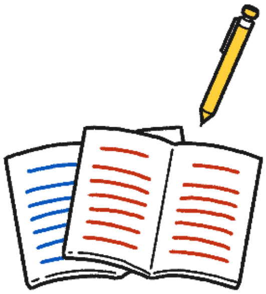

How to use this decision aid?

This decision aid is meant for discussion with the healthcare provider and determination of the future treatment. You can take this aid home, read it carefully, and discuss it with your family members; therefore, the future treatment plan may be contemplated together.

This decision aid should be read carefully

Answers should be marked using a circle or the memo fields should be filled.

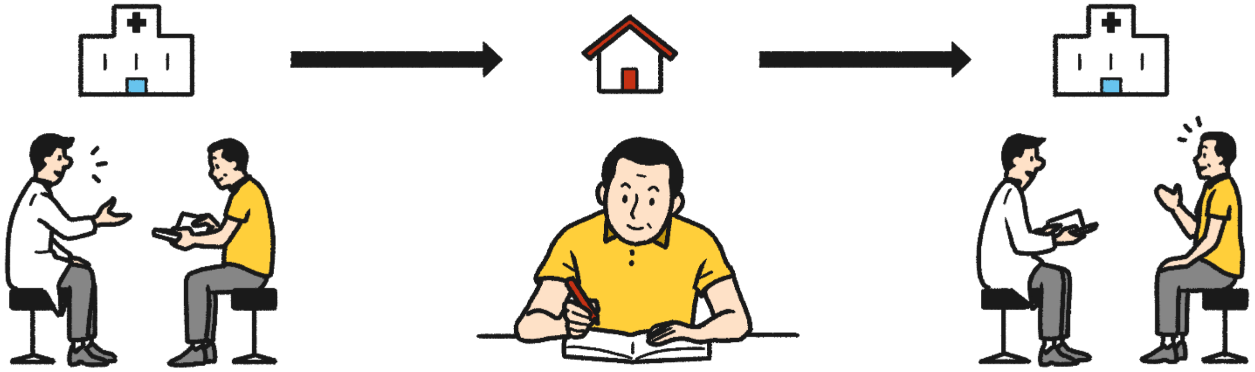

During consultation

・Your current condition will be explained

・Treatment options will be reviewed

During consultation

・The circled items and the contents of the memo should be discussed.

・Further treatment should be planned.

※If we can't decide at once, it should be brought back for further consideration.

What is schizophrenia?

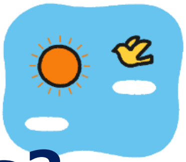

Schizophrenia often begins in adolescence or young adulthood, with symptoms such as hallucinations and delusions, greatly affecting social and daily life.

The treatment goal is to live each day with hope (recovery). Both medication and psychosocial treatments are considered essential to achieve recovery.

The mainstay of medication treatment is antipsychotics, which are highly effective in suppressing hallucinations and delusions. Ideally, antipsychotics should be taken as a single agent and in appropriate doses. Monotherapy refers to the use of only one antipsychotic. The appropriate dosage varies from person to person, and this aid uses the chlorpromazine equivalent, which is ≤600 mg/day.

This aid is intended for those who wish to reduce two antipsychotics to one or a medication with a chlorpromazine equivalent of ≥600 mg/day to <600 mg/day.

What this aid does NOT address or recommend

- **This aid does NOT deal with those who do not have a confirmed diagnosis of schizophrenia.**

Schizophrenia is an illness in which hallucinations and delusions persist for 6 months or longer if untreated.

It is generally divided into the following phases: prodromal (when symptoms first appear), acute (when symptoms are active), and residual (when symptoms subside with antipsychotics). This decision aid deals with **those in the residual phase.**

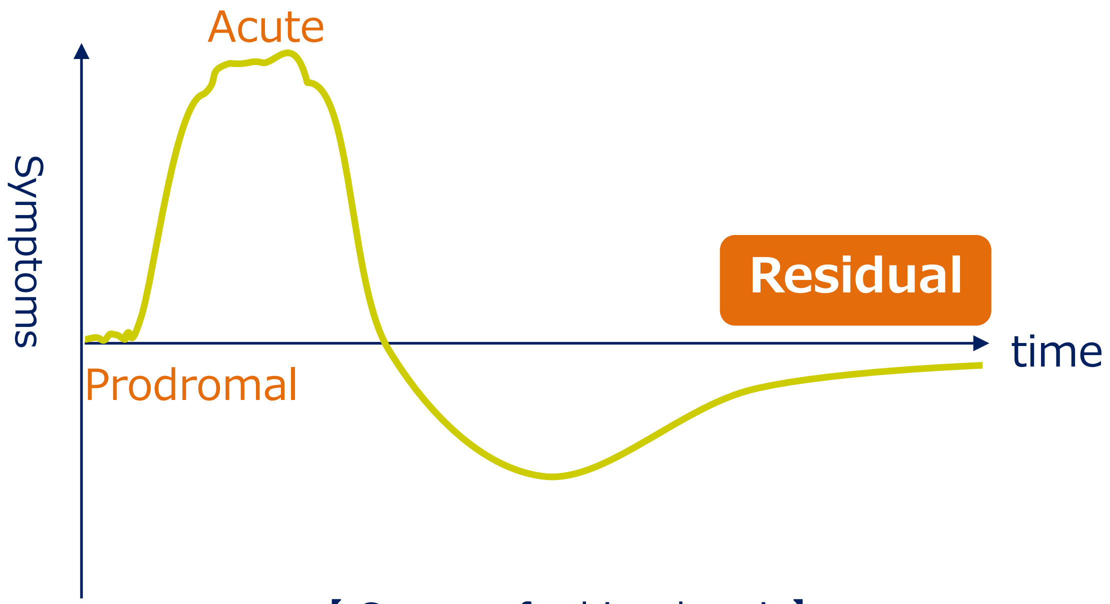

【Course of schizophrenia】

What this aid does NOT address or recommend

- **Drug reduction is NOT recommended for those with unsettled symptoms.**

  Reducing the dose or type of antipsychotic worsens the symptoms of schizophrenia at a certain rate. Therefore, this aid does not recommend reducing the dose or type of antipsychotic for those whose current illness symptoms have not settled.

- **Taking more than three antipsychotics is NOT recommended.**

  Taking more than three antipsychotics at once is not common, and research is scarce on the subject. Therefore, it is not covered in this aid; if you are taking more than three antipsychotics and would like to reduce the dose or type, please discuss with your doctor why you need more than three antipsychotics.

- **Not taking antipsychotics (withdrawal or abstinence) is NOT recommended.**

  Not taking antipsychotics (withdrawal or abstinence) often exacerbates medical conditions and makes recovery difficult. Therefore, it is NOT recommended in this aid.

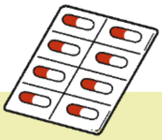

What this aid does NOT address or recommend

- **This aid does NOT deal with drugs other than antipsychotics.**

In addition to antipsychotics, other groups of medications are used in psychiatry, including sleeping pills, anxiolytics, antidepressants, anticonvulsants, and mood stabilizers (to improve mood swings in manic-depressive episodes). These are not medications that improve the original symptoms of schizophrenia, and their concomitant use is not addressed in this aid. If you are taking psychiatric medications other than antipsychotics and would like to reduce the dose or type of medication, please discuss with your doctor why you need these medications.

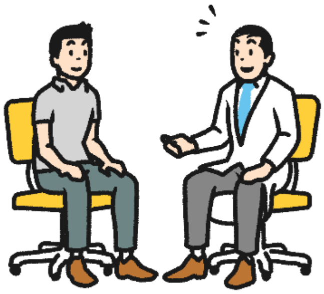

Next, we will look at future treatment options for those who are eligible for this aid. ☞

Further treatment options

There are two patterns of further treatment options.

A For antipsychotic monotherapy,

Option 1

'Reducing the dose of one antipsychotic'

Ex. Reducing from eight to four tables

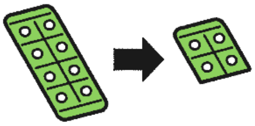

Option 2

'Taking one antipsychotic at the same dose without reducing the dose'

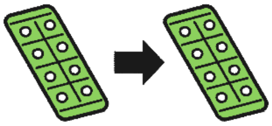

B For the combined use of two antipsychotic drugs,

Option 1

'Reducing the number of antipsychotics from two to one' (Shifting to monotherapy)

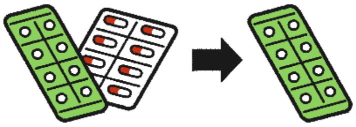

Option 2

'Continuing two antipsychotic drugs without reducing the number' (Continuing polypharmacy)

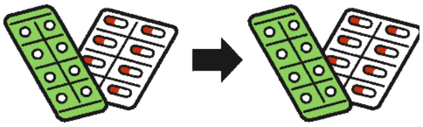

We will explain A and B separately.

A Further treatment options

Step 1 Know the characteristics of each option

A For antipsychotic monotherapy,

Option 1

'Reducing the dose of one antipsychotic'

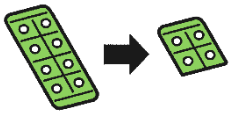

What to do if choosing this option:

・Implementing coping strategies for recovery

・Reducing the dose of one antipsychotic

Option 2

'Taking one antipsychotic at the same dose without reducing the dose'

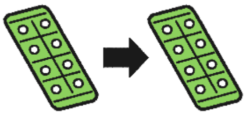

What to do if choosing this option:

・Implementing coping strategies for recovery

・Taking one antipsychotic at the same dose without reducing the dose

See Appendices for the related information:

Appendix1 What is the goal (recovery) in schizophrenia treatment?
Appendix2 What you can do in your daily life to achieve recovery
Appendix3 Chlorpromazine equivalent dose conversion table
Appendix4 Knowing about side effects
Appendix5 Types of antipsychotics and related side effects

We will look at what you need to know, depending on the option you choose. ☞

A Option 1

‘Reducing the dose of one antipsychotic’

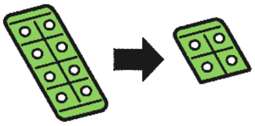

It is important to continue to take antipsychotics. This is because using antipsychotics can alleviate symptoms of mental health conditions, such as hallucinations and delusions, and help progress treatment for recovery.

While a stable physical and mental condition is desirable for recovery, the amount of medication used may become excessive, or the types of medication used may become numerous (pattern B).

Various factors such as the condition and course of the illness affect this. However, for those with stable symptoms but severe side effects, reducing the amount of medication may reduce the side effects.

A Option 1

‘Reducing the dose of one antipsychotic’

- Continuation -

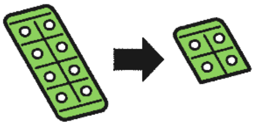

Currently, it is unclear how medication should be reduced. Please refer to the pros and cons described later (page 14) and the appendices at the end of this decision aid, and start discussing with your doctor how to reduce medication and at what pace.

You should know what medication you are currently taking and how much. If you are concerned, please refer to Appendix 3 at the end of this aid, and check with your doctor.

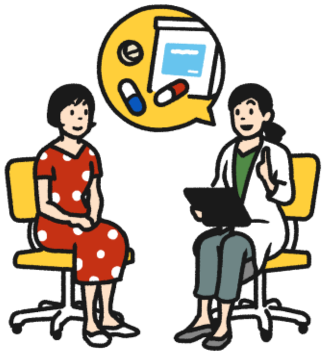

A Option 2

‘Taking one antipsychotic at the same dose without reducing the dose’

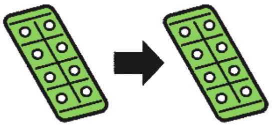

Those who are interested in this option may be those who are currently in stable physical and mental conditions, making some progress for recovery or calmly going about your daily lives.

Continuing antipsychotics is important. Moreover, to properly understand the effects and side effects of the medication, an appropriate dose of the antipsychotic should be used for treatment.

Some may be on a very high dosage because of the course of treatment. However, while individuals may stabilize the physical and mental conditions, they may also cause side effects. The risk of side effects may increase.

A **Option 2**

‘Taking one antipsychotic at the same dose without reducing the dose’

- Continuation -

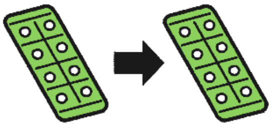

If you wish to continue your treatment with the current medication dosage, you may consider referring to the pros and cons described in page 14 and the appendices at the end of this decision aid as a reference. You could also discuss the dosage and medication form (tablet, powder, liquid, injection, etc.) with your doctor.

It is essential to know the current medication and dosage you are taking. If you are unsure, you may refer to Appendix 3 at the end of this decision aid, and confirm it with your doctor.

Next, let's compare the pros and cons of each option ☞

A Pros and cons of each option

Step 2 Compare the pros and cons of each option

	Option 1<br>**Reducing the dose of one antipsychotic**	Option 2<br>**Taking one antipsychotic at the same dose without reducing the dose**
**Pros** ☺	● Side effects such as extrapyramidal symptoms and autonomic nervous system dysfunction may decrease.<br>● Side effects that increase as the amount of medication increases, such as cognitive dysfunction and sudden cardiac death, may decrease.<br>● Reduced side effects may make it easier to move the body.<br>● The cost of medication may decrease.	● No changes in symptoms<br>● Can maintain a stable condition
**Cons** ☹	● Symptoms may worsen.<br>● Worsening symptoms may require hospitalization.	● Side effects such as extrapyramidal symptoms and automatic nervous dysfunction may occur.<br>● Some side effects increase as the amount of medication increases, such as cognitive dysfunction and sudden cardiac death.<br>● The cost of medication may be high.

A Consequences of each option

Step 3 Compare the consequences of each option

Medication continuation is crucial in the schizophrenia treatment. The research results[1-5)] show how each treatment option can affect medication continuation. The percentage of those using antipsychotics who can continue treatment without problems is shown for each option selected.

	**Option 1**<br>**Reducing the dose of one antipsychotic**	**Option 2**<br>**Taking one antipsychotic at the same dose without reducing the dose**
1 – 12 months	Of the 151 individuals, 121 (80.1%) could continue antipsychotic treatment without problems when reducing the dose of one antipsychotic	Of the 171 individuals, 105 (89.7%) could continue antipsychotic treatment without problems when taking one antipsychotic at the same dose without reducing the dose

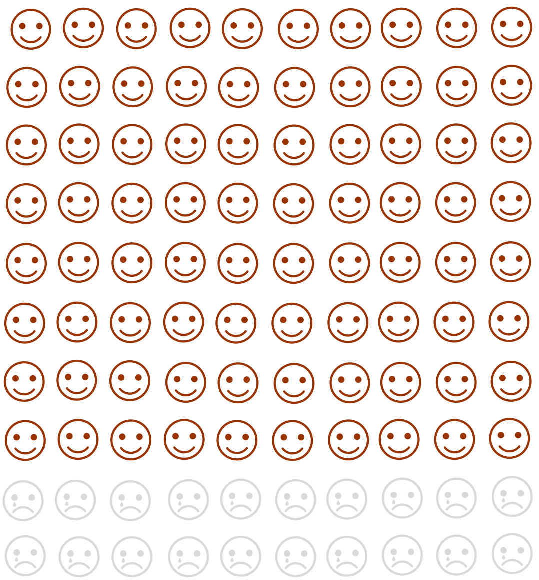

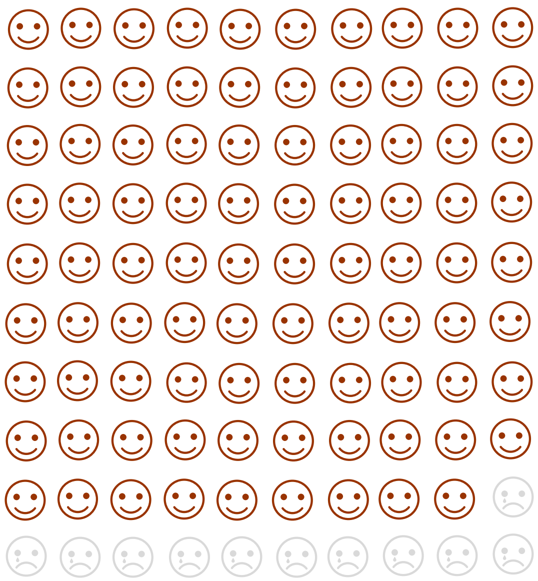

There was no statistically significant difference in the percentage of people in both groups.

A What is important to you?

Step 4 Organize what is important to you.

Below we have listed the main reasons for choosing each option. The importance of each of these to you should be analysed. The numbers that are applicable should be encircled with your importance.

Option 1 Reason for 'Reducing the dose of one antipsychotic'

	Not important					Important
Side effects may be decrease.	0	1	2	3	4	5
Reduced side effects may make it easier to move the body	0	1	2	3	4	5
Other (please state freely)						
・	0	1	2	3	4	5
・	0	1	2	3	4	5
・	0	1	2	3	4	5

Option 2 Reason for 'Taking one antipsychotic at the same dose without reducing the dose'

	Not important					Important
No changes in symptoms	0	1	2	3	4	5
Can maintain a stable condition	0	1	2	3	4	5
Other (please state freely)						
・	0	1	2	3	4	5
・	0	1	2	3	4	5
・	0	1	2	3	4	5

A

Preparing for a consultation discussion

Step 5 Preparing for the discussion with the doctor

Based on the thoughts and weightage, we will discuss **Option1** ‘Reducing the dose of one antipsychotic’ and **Option2** ‘Taking one antipsychotic at the same dose without reducing the dose’ in the consultation.

Please note down any current feelings and thoughts, including any questions or concerns.

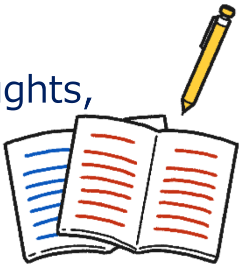

B Further treatment options

Step 1 Know the characteristics of each option

B For the combined us of two antipsychotic drugs,

Option 1

'Reducing the number of antipsychotics from two to one' (Shifting to monotherapy)

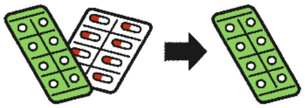

What to do if choosing this option:

・Implementing coping strategies for recovery

・Reducing the number of antipsychotic from two to one

Option 2

'Continuing two antipsychotic drugs without reducing the number' (Continuing polypharmacy)

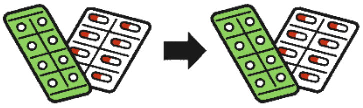

What to do if choosing this option:

・Implementing coping strategies for recovery

・Continuing two antipsychotic drugs without reducing the number

See Appendices for the related information:

Appendix1 What is the goal (recovery) in schizophrenia treatment?

Appendix2 What you can do in your daily life to achieve recovery

Appendix3 Chlorpromazine equivalent dose conversion table

Appendix4 Knowing about side effects

Appendix5 Types of antipsychotics and related side effects

We'll look at what you need to know, depending on the option you choose. ☞

B **Option 1**

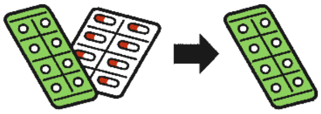

'Reducing the number of antipsychotics from two to one' (Shifting to monotherapy)

Continuation of antipsychotics is important. To properly understand the effects and side effects of the antipsychotics you are taking, it is fundamental to take one type of antipsychotics. Therefore, when two types of antipsychotics are used following the previous condition and treatment course, consolidating to one type of antipsychotics may be required.

However, this approach has pros and cons, as described later (page 22). Additionally, the method and pace of reducing the number of types of antipsychotics used can vary depending on the medication being used. Please refer to the appendices in this aid, and consult with your doctor to confirm and discuss what should be taken into consideration.

＊Use of three or more antipsychotics＊

No specific evidence verifies the benefits or risks of using three or more antipsychotics in treatment, and unforeseen risks may exist. Therefore, sufficient consultation should be made with healthcare providers, including your doctor, regarding further medication treatment.

B **Option 2**

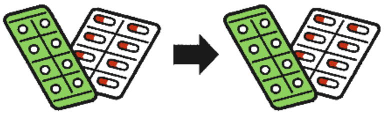

‘Continuing two antipsychotic drugs without reducing the number’ (Continuing polypharmacy)

Those who are interested in this option may be those who are currently in stable physical and mental conditions, making some progress for recovery, or calmly going about their daily lives.

Continuing antipsychotics is important. Moreover, to properly understand the effects and side effects of the medication, it is important to treat with one antipsychotic.

Some may be on two types of antipsychotics because of the course of treatment. However, while individuals may help stabilise psychiatric symptoms, if side effects occur, determining which medication is responsible may be difficult.

B **Option 2**

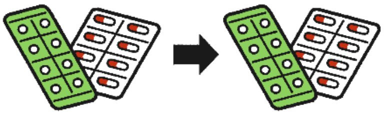

‘Continuing two antipsychotic drugs without reducing the number’ (Continuing polypharmacy) - continuation

Even if you wish to continue treatment with two antipsychotics, you may want to discuss with your doctor what precautions to take in continuing with two antipsychotic drugs, referring to the pros and cons (page 22) and the appendix at the end of this document, as discussed later. You should know what medications you are currently using and at what dosage. If you are concerned about this, please refer to Appendix 3 at the end of this document, and check with your doctor.

＊Use of three or more antipsychotic medications＊

There is no specific evidence to verify the benefits or risks of using three or more antipsychotic drugs in treatment, and there may be unforeseen risks. Therefore, it is necessary to have sufficient consultation with healthcare providers, including your doctor, regarding further medication treatment.

Next, let's compare the pros and cons of each option ☞

B Pros and cons of each option

Step 2 Compare the pros and cons of each option

	Option 1<br>**Shifting to monotherapy**	Option 2<br>**Continuing polypharmacy**
**Pros** ☺	● Side effects such as extrapyramidal symptoms and autonomic nervous system dysfunction may decrease.<br>● Side effects that increase as the amount of medication increase, such as cognitive dysfunction and sudden cardiac death, may decrease.<br>● Reduced side effects may make it easier to move the body<br>● Medication has become simpler and easier to take.<br>● The cost of medication may decrease	● No changes in symptoms<br>● Can maintain a stable condition
**Cons** ☹	● Transition to monotherapy may not go smoothly<br>● Symptoms may worsen<br>● Worsening symptoms may require hospitalization	● There may be concerns about side effects such as extrapyramidal symptoms, and automatic nervous dysfunction.<br>● There may be concerns about side effects that are known to increase as the mount of medication increase, such as cognitive dysfunction and sudden cardiac death<br>● There may be a cost of medication<br>● The risk of side effects may increase with continuing two antipsychotics.

B Consequences of each option

Step 3 Compare the consequences of each option

Medication continuation is crucial in the schizophrenia treatment. The research results[6-11] show how each treatment option can affect medication continuation. The percentage of those using antipsychotics who can continue treatment without problems is shown for each option selected.

	Option 1<br>**Shifting to monotherapy**	Option 2<br>**Continuing polypharmacy**
1 – 12 months	Of the 177 individuals, 120 (67.8%) could continue antipsychotic treatment without problems when shifting to monotherapy.<br>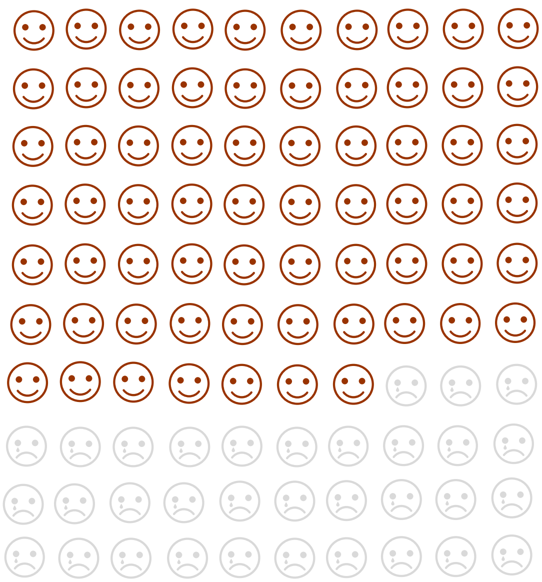	Of the 164 individuals, 140 (85.4%) could continue antipsychotic treatment without problems when continuing the current two antipsychotics.<br>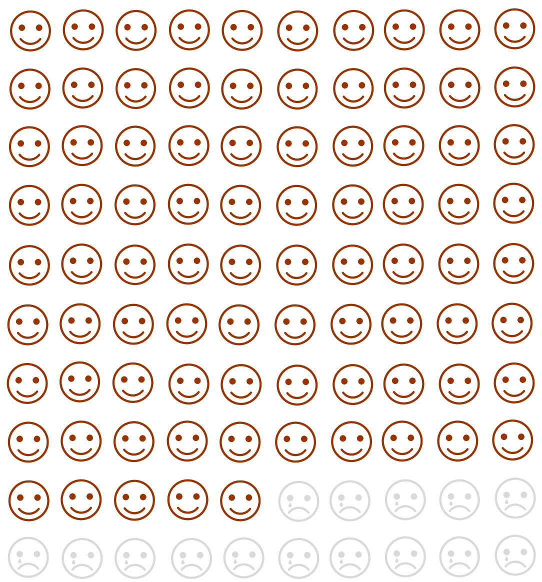

There was a statistically significant difference in the percentage of people in both groups.

B What is important to you?

Step 4 Organize what is important to you.

Below we have listed the main reasons for choosing each option. The importance of each of these to you should be analysed. The numbers that are applicable should be encircled with your importance.

Option 1 Reason for 'Shifting to monotherapy'

	Not important					Important
Side effects may be decrease.	0	1	2	3	4	5
Reduced side effects may make it easier to move the body	0	1	2	3	4	5
Other (please state freely)						
·	0	1	2	3	4	5
·	0	1	2	3	4	5
·	0	1	2	3	4	5

Option 2 Reason for 'Continuing polypharmacy'

	Not important					Important
No changes in symptoms	0	1	2	3	4	5
Can maintain a stable condition	0	1	2	3	4	5
Other (please state freely)						
·	0	1	2	3	4	5
·	0	1	2	3	4	5
·	0	1	2	3	4	5

B

Preparing for a consultation discussion

Step 5 Preparing for the discussion with the doctor

Based on the thoughts and weightage, we will discuss **Option1** 'Shifting to monotherapy' and **Option2** 'Continuing polypharmacy'.

Please note down any current feelings and thoughts, including any questions or concerns.

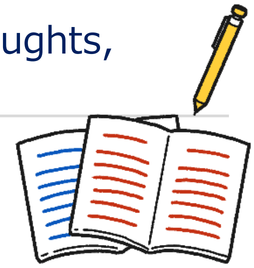

Appendix1

What is the goal (recovery) in schizophrenia treatment?

Appendix2

What you can do in your daily life to achieve recovery

Appendix3

Chlorpromazine equivalent dose conversion table

Appendix4

Knowing about side effects

Appendix5

Types of antipsychotics and related side effects

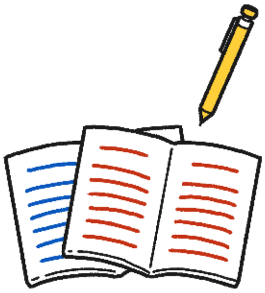

Appendix1

What is the goal (recovery) in schizophrenia treatment?

> ***Recovery is a deeply personal, unique process of changing one's attitudes, values, feelings, goals, skills and/or roles. It is a way of living a satisfying, hopeful, and contributing life even with limitations caused by the illness.*** *(W. A. Anthony)*

Do you know the concept of "recovery" in the treatment of schizophrenia? While living with an undesirable condition, it is important to think about what you want to become and what you aspire to achieve. The idea of recovery may be helpful in this regard.

There are various approaches to recovery, but it is generally considered to have two aspects: clinical and personal, as shown in the diagram on the next page. This means that stabilizing the illness, improving and maintaining symptoms and function, and approaching the life that the person desires are equally important.

Clinical recovery, according to Liberman's definition, involves achieving sustained remission of symptoms, engaging in vocational or educational activities for at least half a day, living independently (both financially and in terms of treatment), and building social relationships (with at least one friend per week) for a duration of at least 2 years.

Appendix1

What is the goal (recovery) in schizophrenia treatment?
- Continuation -

However, that is not the only aspect that is important. The process of finding meaning and purpose in life for oneself is called personal recovery. It is desirable to consult and discuss with healthcare providers and significant people and work for recovery together, even if it occasionally involves difficulties and worries.

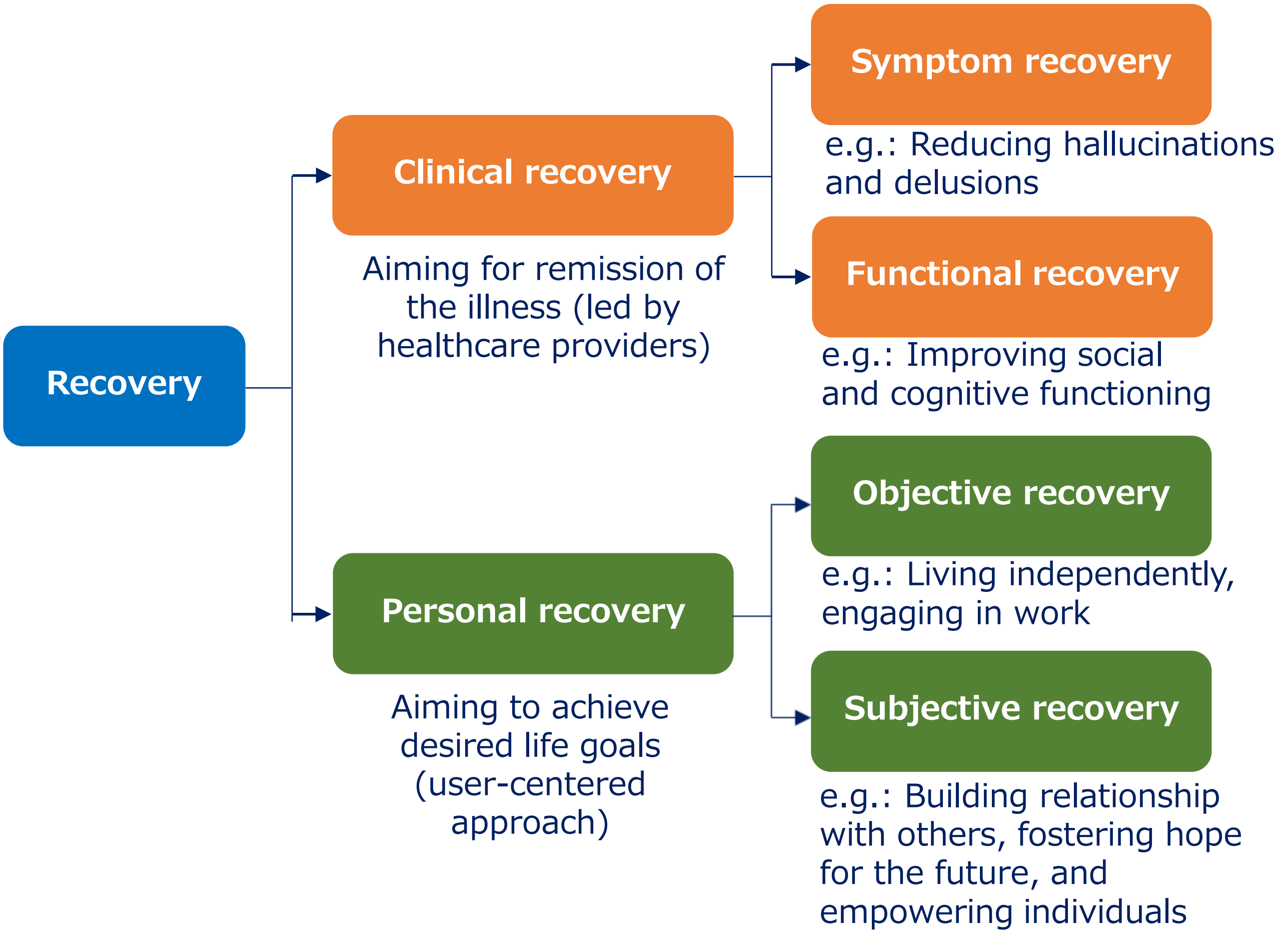

Yamaguchi S: Igakuno Ayumi, 261(10), 941-8, 2017 [15)]

※ key components of personal recovery, Leamy et al., 2011 [16)]
1. Connectedness, 2. Hope and optimism, 3. Identity,
4. Meaning in life, and 5. Empowerment

Appendix2

What you can do in your daily life to achieve recovery

Here, we have provided examples of what you can do in daily life to work for recovery. Please feel free to use them as a reference.“

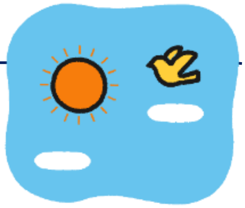

- Having a vision for the life one wants
- Having enjoyable moments
- Feeling a sense of control over one's life
- Being able to articulate hopes and aspirations
- Feeling useful to others
- Maintaining a regular routine
- Getting sufficient sleep
- Having a place to be during the day
- Having someone to talk to (friends, family, cares, etc.) and a sense of belonging
- Knowing and accessing necessary supports for daily living
- Knowing and accessing necessary supports for treatment
- Knowing and accessing necessary supports for social participation, such as employment
- Understanding one's illness
- Understanding one's own condition and symptoms
- Knowing signs of worsening condition and how to respond to them
- Deciding whom to contact in case of emergency

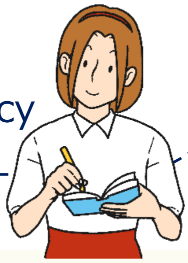

Appendix3 Chlorpromazine equivalent dose conversion table

This is a chart of medication doses that are considered equivalent to 600 mg/day of chlorpromazine (roughly the same strength). If you are currently taking a medication at a higher dose than what is listed here, you are within the scope of this decision aid.

1. Oral drugs

Nonproprietary name	Typical Japanese trade name	Equivalent Conversion Dose Amount
Asenapine	Sycrest	15mg*
Aripiprazole	Abilify	30mg
Olanzapine	Zyprexa	20mg
Quetiapine	Seroquel	750mg
Clozapine	Clozaril	400mg
**Chlorpromazine**	**Contomin**	**600mg**
Sultopride	Barnetil	1200mg*
Sulpiride	Dogmatil	800mg
Zotepine	Lodopin	300mg
Tiapride	Gramalil	600mg*
Timiperone	Tolopelon	7.8mg*
Nemonapride	Emilace	27mg*
Paliperidone	Invega	9mg
Haloperidol	Serenace	10mg
Pimozide	Orap	8mg
Fluphenazine	Flumezin	12mg
Brexpiprazole	Rexulti	No information
Blonanserin	Lonasen	24mg*
Propericiazine	Neuleptil	120mg*
Bromperidol	Impromen	12mg*
Perphenazine	PZC	30mg
Perospirone	Lullan	48mg*
Risperidone	Risperdal	6mg
Levomepromazine	Levotomin	400mg

Revised Table by Gardner et al.[12)]

Appendix3

Chlorpromazine equivalent dose conversion table - Continuation -

2. Injection

Nonproprietary name	Typical Japanese trade name	Equivalent Conversion Dose Amount
Aripiprazole long-acting injection	Abilify long-acting injection	600mg Every 4 weeks*
Paliperidone Palmitate	Xeplion	112.5mg Every 4 weeks*
Haloperidol Decanoate	Halomonth	150mg Every 4 weeks
Fluphenazine Decanoate	Fludecasin	50mg Every 4 weeks
Risperidone long-acting injection	Risperdal Consta	50mg Every 2 weeks

Revised Table by Gardner et al.[12)]

Note: This table was created based on the conversion table [12)] reported by Gardner et al. (2010). However, for drugs marked with an asterisk (*), since Gardner et al. (2010) provided no information, values based on the conversion table by Inagaki and Inada (2015,2017) [13,14)] have been referred.

Appendix 4 Knowing about side effects

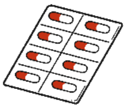

Antipsychotics are crucial in schizophrenia treatment. However, they may have side effects that can manifest in the symptoms listed below. If you experience any of the following side effects, please consult your doctor.

- Excessive increase in appetite/weight gain
- Persistent thirst/drinking a lot of water
- Constipation/difficulty in urination
- Inability to focus eyes/blurred vision
- Absence of menstruation/breast milk production
- Erectile dysfunction/decreased libido
- Inability to sit still
- Restlessness in the legs, feeling compelled to move around
- Involuntary movements of the mouth or hands/trembling of the hands or fingers
- Tongue twisting, difficulty speaking clearly
- Increased salivation or drooling
- Muscle stiffness, difficulty in body movements
- Excessive drowsiness, constant fogginess/dizziness upon standing

Please remember to consult the healthcare providers regarding these side effects.

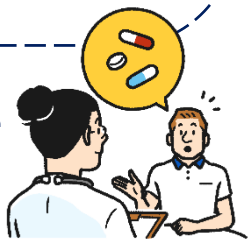

Appendix5 Types of antipsychotics and related side effects

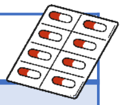

Side effects	Frequency	Antipsychotics
Constipation, dry mouth, and other symptoms that make it difficult to abruptly discontinue the drug	High	Clozapine[1], Chlorpromazine
	Middle	Olanzapine, Quetiapine, Paliperidone, Haloperidol, Risperidone
Increased drowsiness	High	Olanzapine, Quetiapine, Clozapine[1], Chlorpromazine
	Middle	Paliperidone, Haloperidol, Risperidone Lurasidone
Weight gain	High	Olanzapine, Quetiapine, Clozapine[1], Chlorpromazine, Paliperidone, Risperidon
	Middle	Asenapin, Sulpiride, Haloperidol, Brexpiprazole
Akathisia (restlessness)[2]	High	Haloperidol
	Middle	Asenapin, Aripiprazole, Chlorpromazine Sulpiride, Brexpiprazole, Risperidone Lurasidone
Parkinson’s-like symptoms[3]	High	Chlorpromazine, Haloperidol
	Middle	Sulpiride, Paliperidone, Risperidone, Lurasidone
Decreased blood pressure	High	Quetiapine, Clozapine[1], Chlorpromazine Paliperidone, Risperidone
	Middle	Olanzapine, Haloperidol
Increased prolactine levels[4]	High	Chlorpromazine, Sulpiride, Paliperidone Haloperidol, Risperidone
	Middle	Asenapin, Olanzapine

Revised Table in The Maudsley Prescribing Guidelines in Psychiatry, 13th Edition

See Appendix 1, etc. for typical Japanese trade names

1. Used only for those with treatment-resistant symptoms (such as lack of improvement even after using two or more adequate antipsychotic medications for a sufficient duration).
2. It mainly induces a restless or agitated sensation in the legs, or "restless legs syndrome”.
3. Parkinson's-like symptoms include slowed movements, decreased voice volume, reduced facial expressions, unsteady gait, shuffling gait (small steps), difficulty initiating the first step, hand tremors, inability to stop or sudden bursts of running, and stiffness in the limbs.
4. Increased levels of the hormone prolactin can lead to menstrual abnormalities in women and reduced libido or breast enlargement in men.

In addition to those listed in the table, other antipsychotic medications can be used for schizophrenia in Japan. Please ask your doctor about the characteristics of these medications.

Conclusion

● To determine the right coping and treatment methods that are best for you

Each treatment option has its pros and cons. This decision aid is designed to help you understand these options well, discuss them with your healthcare provider while determining what is important to you, and make the choices that are right for you.

● Development of the decision aid

This decision aid was developed based on the responses and opinions of people who had been taking medication treatment for schizophrenia The decision aid has also been checked by psychiatric specialists. In addition, this aid was developed by research grants from the Ministry of Health, Labor and Welfare of Japan (H29-精神-一般-001, 19GC1012).

● Updating the decision aid

This decision aid will be reviewed and updated as necessary.

The information provided here is intended to guide you in identifying the right coping or treatment method for you while you are consulting with a healthcare professional; however, this decision aid is not intended to replace the advice provided by the healthcare professional.

References
1) Yamanouchi Y, Sukegawa T, Inagaki A, Inada T, Yoshio T, Yoshimura R, Iwata N. Evaluation of the individual safe correction of antipsychotic agent polypharmacy in Japanese patients with chronic schizophrenia: validation of safe corrections for antipsychotic polypharmacy and the high-dose method. Int J Neuropsychopharmacol. 2014;18(5).
2) Uchida H, Suzuki T, Yamazawa R, Tomita M, Nemoto T, Kimura Y, Watanabe K, Imasaka Y, Kashima H. Reducing the dose of antipsychotic agents ameliorates visual hypersensitivity attack: an ideal treatment option in terms of the adverse effect. J Clin Psychopharmacol. 2006; 26(1):50-5.
3) Volavka J, Cooper TB, Czobor P, Lindenmayer JP, Citrome LL, Mohr P, Bark N. High-dose treatment with haloperidol: the effect of dose reduction. J Clin Psychopharmacol. 2000; 20(2):252-6.
4) Hirschowitz J, Hitzemann R, Piscani K, Burr G, Frecska E, Culliton D, Mann M, Curtis C. The Dose Reduction in Schizophrenia (DORIS) Study: a final report. Schizophr Res. 1997;23(1):31-43.
5) Newcomer JW, Riney SJ, Vinogradov S, Csernansky JG. Plasma prolactin and homovanillic acid as markers for psychopathology and abnormal movements after neuroleptic dose decrease. Psychopharmacol Bull. 1992;28(1):101-7.
6) Essock SM, Schooler NR, Stroup TS, McEvoy JP, Rojas I, Jackson C, Covell NH. Effectiveness of Switching From Antipsychotic Polypharmacy to Monotherapy. Am J Psychiatry. 2011;168(7):702–8.
7) Constantine RJ, Andel R, McPherson M, Tandon R. The Risks and Benefits of Switching Patients with Schizophrenia or Schizoaffective Disorder from Two to One Antipsychotic Medication: A Randomized Controlled Trial. Schizophrenia Research. 2015;166(1–3):194–200.
8) Hori H, Yoshimura R, Katsuki A, Sugita AI, Atake K, Nakamura J. Switching to Antipsychotic Monotherapy Can Improve Attention and Processing Speed, and Social Activity in Chronic Schizophrenia Patients. J Psychiatr Res. 2013;47(12):1843–48.
9) Repo-Tiihonen E, Hallikainen T, Kivistö P, Tiihonen J. Antipsychotic Polypharmacy in Clozapine Resistant Schizophrenia: A Randomized Controlled Trial of Tapering Antipsychotic Co-Treatment. Mental Illness. 2012; 4(1):1–4.
10) Borlido C, Remington G, Graff-Guerrero A, Arenovich T, Hazra M, Wong A, Daskalakis ZJ, Mamo DC. Switching from 2 Antipsychotics to 1 Antipsychotic in Schizophrenia: A Randomized, Double-Blind, Placebo-Controlled Study. J Clin Psychiatry. 2016;77(1):e14-20.
11) Yoon HW, Lee JS, Park SJ, Lee SK, Choi WJ, Kim TY, Hong CH, Seok JH, Park IH, Son SJ, Roh D, Kim BR, Lee BO. Comparing the Effectiveness and Safety of the Addition of and Switching to Aripiprazole for Resolving Antipsychotic-Induced Hyperprolactinemia: A Multicenter, Open-Label, Prospective Study. Clin Neuropharmacol. 2016;39(6):288–94.
12) Gardner DM, Murphy AL, O'Donnell H, Centorrino F, Baldessarini RJ. International consensus study of antipsychotic dosing. Am J Psychiatry. 2010;167:686-93.
13) Inagaki A, Inada T : Japanese Journal of Psychopharmacology 20 : 89-97, 2017
14) Inagaki A, Inada T : Japanese Journal of Psychopharmacology 18 : 1475-1480, 2015
15) Yamaguchi S, Kumakura Y : Journal of Clinical and Experimental Medicine 261 : 941-948, 2017
16) Leamy M, Bird V, Le Boutillier C, Williams J, Slade M. Conceptual framework for personal recovery in mental health: systematic review and narrative synthesis. Br J Psychiatry. 2011;199:445-52.
17) Taylor DM, et al./Uchida H et al. trans. The Maudsley Prescribing Guidelines in Psychiatry 13th Edition. Wiley Publishing Japan.2019.

Developed by Antipsychotics Group in
Psychotropic Drugs Exit Strategy Manual Research Group

Developed : March 2023 Scheduled to be updated : March 2025

This decision aid is based on the Guidelines for
Psychotropic Drug Discontinuation Strategies

©2023, Psychotropic Drugs Exit Strategy Manual Research Group
