# Supplementary material for: Development and acceptability testing of a decision aid for considering whether to reduce antipsychotics in individuals with stable schizophrenia
Source: Neuropsychopharmacol Rep. 2023 Jul 14;43(3):391–402. doi: 10.1002/npr2.12366 (PMC10496039; doi:10.1002/npr2.12366)
Supplement: Supplementary file 7 — Appendix S7 [file NPR2-43-391-s005.pdf]

Appendix 4. International Patient Decision Aid Standards criteria met by current decision aid (Joseph-Williams et al., 2014)

Item	1. Qualifying Criteria	2. Certification Criteria	3. Quality Criteria
Information	Describes the health condition or problem for which decision is required [a]<br>Explicitly states decision that needs to be considered [a]<br>Describes the options available for the index decision [a]<br>Describes positive features of each option [a]<br>Describes negative features of each option [a]	Shows the negative and positive features of options with equal detail [a]	Describes the natural course of the health condition or problem if no action is taken [a]<br>Makes it possible to compare the positive and negative features of available options [a]
Probabilities			Provides information about outcome probabilities associated with the options [a]<br>Specifies the defined group of patients for whom the outcome probabilities apply [a]<br>Specifies the event rates for outcome probabilities [a]<br>Allows the user to compare outcome probabilities across options using the same time period [a]<br>Allows the user to compare outcome probabilities across the same denominator [a]<br>Provides more than 1 way of viewing the probabilities (e.g., words, numbers, diagrams) [a]
Values	Describes what it is like to experience consequence of the options [a]		Asks patients to think about which positive and negative features of options matter most to them [a]
Guidance			Provides a step-by-step way to make a decision [a]<br>Includes tools like worksheets or lists of questions to use when discussing options with a practitioner [a]
Development			Development process included a needs assessment with clients or patients [a]<br>Development process included a needs assessment with health professionals [a]

Appendix 4. *Continued*

Item	1. Qualifying Criteria	2. Certification Criteria	3. Quality Criteria
			Development process included review by clients/patients not involved in producing the decision support intervention [a]
			Development process included review by professionals not involved in producing the decision support intervention [a]
			Field tested with patients who were facing the decision [b]
			Field tested with practitioners who counsel patients who face the decision [b]
Evidence		Provides citations to the evidence selected [a]	Describes how research evidence was selected or synthesized [a]
		Provides a production or publication date [a]	Describes the quality of the research evidence used [a]
		Provides information about the update policy [a]	
		Provides information about the levels of uncertainty around the event or outcome probabilities [a]	
Disclosure		Provides information about the funding source used for development [a]	Includes authors'/developers' credentials or qualifications [a]
Plain Language			Reports readability levels [a]
Evaluation		Describes what the test is designed to measure [b]	Evidence improved match between preferences of the informed patient and the option chosen [b]
			Evidence patient decision aid helps patients improve their knowledge about options' features [b]

a. Criteria met by the developed decision aid

b. Criteria to be met with effectiveness testing, not applicable for the current decision aid
